# Minimizing the risk of allo-sensitization to optimize the benefit of allogeneic cardiac-derived stem/progenitor cells

**DOI:** 10.1038/srep41125

**Published:** 2017-01-24

**Authors:** Hocine R. Hocine, Hicham E. L. Costa, Noemie Dam, Jerome Giustiniani, Itziar Palacios, Pascale Loiseau, Armand Benssusan, Luis R. Borlado, Dominique Charron, Caroline Suberbielle, Nabila Jabrane-Ferrat, Reem Al-Daccak

**Affiliations:** 1 Institut National de la Santé et de la Recherche Médicale (INSERM) UMRS-976, Université Paris Diderot, Hôpital Saint-Louis, Paris, France; 2 HLA et Medicine, Hôpital Saint Louis, Paris, France; 3 INSERM UMR 1043, Centre National Recherche Scientifique UMR 5282, Université Toulouse III Paul Sabatier, Toulouse, France; 4 Cortherapix, S.L., Madrid, Spain; 5 Laboratoire d’Immunologie et d’Histocompatibilité, Hôpital Saint Louis, Paris, France

## Abstract

Allogeneic human cardiac-derived stem/progenitor cells (hCPC) are currently under clinical investigation for cardiac repair. While cellular immune response against allogeneic hCPC could be part of their beneficial-paracrine effects, their humoral immune response remains largely unexplored. Donor-specific HLA antibodies (DSA-HLA-I/DSA-HLA-II), primary elements of antibody-mediated allograft injury, might present an unidentified risk to allogeneic hCPC therapy. Here we established that the binding strength of anti-HLA monoclonal antibodies delineates hCPC proneness to antibody-mediated injury. *In vitro* modeling of clinical setting demonstrated that specific DSA-HLA-I of high/intermediate binding strength are harmful for hCPC whereas DSA-HLA-II are benign. Furthermore, the Luminex-based solid-phase assays are suitable to predict the DSA-HLA risk to therapeutic hCPC. Our data indicate that screening patient sera for the presence of HLA antibodies is important to provide an immune-educated choice of allogeneic therapeutic cells, minimize the risk of precipitous elimination and promote the allogeneic reparative effects.

Recent progress in stem/progenitor cell-based cardiac regenerative/reparative therapies has provided new insights into their mode of action as well as into their immune behavior within autologous and allogeneic settings. It is very likely that stem/progenitor cells repair the injured myocardium through constructive paracrine rather than trans-differentiation mechanisms^1^. Nevertheless, both autologous and allogeneic cells need to remain enough time to allow paracrine-associated improvements and promote therapeutic benefit. The largest clinical trial conducted today, the CONCERT-HF (https://clinicaltrials.gov/ct2/show/NCT02501811), has employed autologous cells, which in theory are not recognized by the host immune system and therefore have a more prolonged engraftment than allogeneic cells. However, autologous strategies have encountered certain limitations, and the new era tends to acknowledge allogeneic stem/progenitor cells as being a more realistic and pragmatic cardiac repair strategy^2^,^3^,^4^,^5^.

Currently, a large body of *in vitro* and *in vivo* research indicates that the allogeneic stem/progenitor cells are safe since they activate modulatory rather than deleterious cellular immune reactions^5^,^6^,^7^,^8^,^9^,^10^. This applies to mesenchymal stem cells, cardiosphere-derived cells (CDC), and cardiac-derived stem/progenitor cells (CPC). Moreover, our previous findings also highlight the allogenecity of human CPC as part of the dynamic mechanisms that are critical for the maintenance of sustainable cardiac repair^8^,^10^. All together, these findings prompted the initiation of two clinical trials using allogeneic cardiac stem/progenitor cells: the ALLSTAR (http://clinicaltrials.gov/show/NCT01458405) and the CAREMI (https://clinicaltrials.gov/ct2/show/NCT02439398) in patients with acute myocardial infarction (MI). Yet, a key challenge to using these allogeneic cells for successful clinical practice is their rapid elimination compared to autologous cells^7^,^11^. This might in turn affect their projected paracrine regenerative/reparative actions.

Lessons from allogeneic solid-organ and hematopoietic stem cell (HSC) transplantation indicate that beyond the immune cell-mediated graft destruction, the existence and/or *de-novo* production of donor-specific antibodies against alloantigens (DSA), including the Human Leukocyte Antigens (HLA), are an absolute graft injury factor^12^,^13^,^14^,^15^. Allelic differences at polymorphic HLA loci during blood transfusion, pregnancy, or transplantation induce allogeneic sensitization through the generation of alloantibodies against the class I and class II HLA (DSA-HLA-I and DSA-HLA-II, respectively)^16^,^17^. HLA antibodies are the most frequently encountered alloantibodies in healthy individuals^18^ and act through complement-dependent and -independent mechanisms to provoke humoral graft rejection. They bind and activate the complement through the Fc region, which results in complement-dependent cytotoxicity (CDC) and incites the acute antibody-mediated rejection^19^,^20^. HLA antibodies also activate antibody-dependent cell-mediated cytotoxicity (ADCC) through their Fc region engaging receptors on innate immune cells such as natural killer (NK) cells^21^.

CPCs constitutively express the immunogenic alloantigens, HLA class I (HLA-I). Moreover, a microenvironment rich in growth factors (such as FGF and HGF) and pro-inflammatory cytokines (such as IFNγ and TNFα) would induce the expression of HLA-II on CPCs^8^,^22^. These immunogenic alloantigens would incite the recognition of the infused CPCs by pre-existing DSA-HLA I and II and may also trigger *de-novo* production of these DSA by activated B cells. Hence, DSA-HLA effects are clinically relevant in the context of allogeneic CPC therapies. They might contribute to pre-mature and fast elimination of the transplanted allogeneic cells before the occurrence of their favorable anti-inflammatory modulatory immune response, the allogeneic-driven-benefit.

Studies in swine and rodent models, demonstrated that the immune system reduces the survival of transplanted allogeneic mesenchymal stem cells by eliciting humoral immune response to grafted cells^11^,^23^. Furthermore, xenotransplantation of human embryonic stem cells (hESC) induces a rapid surge of DSA-HLA-I that contribute to immune rejection, whereas HLA-I knockdown remarkably alleviates antibody production and prolongs the survival of hESC^24^. Although the mechanisms involved in humoral allo-rejection of stem cells are still unknown, studies in animal model suggested that CDC and ADCC might be responsible for stem cell elimination *in vivo,* as in the case of organ or cell transplantations^25^.

Sensitive solid-phase assays using Luminex-based technology are the standard practice in allogeneic organ transplantation to detect the presence and identify the specificities of DSA-HLA^26^. These assays determine the mean fluorescence intensity (MFI) of the antibody interaction with HLA-I and -II antigens. The MFI often referred to as “binding strength” is the quantitative and qualitative delineation of DSA-HLA interaction with their targets, and controls the clinical outcome of allogeneic transplantation^27^,^28^. However, the usage of this standard test in CPC therapy is yet to be determined. In fact, the impact of the binding strength as determined by this assay on the outcome of cardiac stem/progenitor cells was never demonstrated.

In this study, we used human cardiac-derived stem/progenitor cells (hCPC) to examine the proneness of cardiac stem/progenitor cells to DSA-HLA induced rejection. hCPC are stem cells with mixed phenotype expressing pluripotency as well as early cardiac lineage transcription factors^8^. We developed a tailored *in vitro* flow cytometry-based assay that allowed us to determine the antigen specificity and the binding strength of circulating DSA-HLA and the antibody-mediated injuries to hCPC. We show that the presence of DSA-HLA-I with high/intermediate binding strength is detrimental for allogeneic hCPC promoting their death. In contrast, DSA-HLA-I with low binding strength or DSA-HLA-II are not. Furthermore, we found a significant correlation between the occurrence of CDC and ADCC by the developed flow cytometry-based assay and the binding strength of DSA-HLA determined by the standard Luminex-based assay. Thus, DSA-HLA-I-sensitization could contribute to the loss of hCPC upon their infusion. A systematic immuno-monitoring of DSA-HLA by Luminex-based assay would provide an immune educated choice of these off-the-shelf allogeneic hCPC, which might permit a prolonged persistence to activate endogenous regeneration and optimize repair impaired heart function.

## Results

### Delineation of the binding strength of the anti-HLA antibody interaction with hCPC by flow cytometry-based assay

In organ transplantation, the clinical relevance and risk of DSA-HLA can be predicted by their binding strength measured by the MFI of their interactions with HLA class I and class II antigens through the Luminex-based assay. Such assay is not yet validated as a tool to measure the binding strength of HLA alloantibody interaction with hCPC or to predict their risk for hCPC transplantation. Therefore, we first used two high-affinity specific anti-HLA-I (W6/32) and -II (anti-HLA-DR L243) monoclonal antibodies (mAb) to develop a flow cytometry-based assay that can assess the capacity of HLA antibodies to interact with hCPC and determine the characteristic of this interaction regardless of the HLA haplotype of the therapeutic cells.

hCPC from six different donors were genotyped for their HLA-I (HLA-A, -B, -C) and HLA-II (HLA-DR) (Supplementary Table 1) then cultured with declining concentrations (10 to 0.05 μg/ml) of each mAb. The reactivity, as MFI, for each antibody concentration was then determined using phycoerythrin (PE)-conjugated anti-IgG secondary antibody and flow cytometry analysis. In theory, the infused hCPC to MI patients few days after injury would operate within post-MI inflammatory environment. They would be primed/stimulated by a variety of growth factors and pro-inflammatory cytokines, such as IFNγ and TNFα that would change their immunological profile without affecting their stem/progenitor properties^8^. Therefore, hCPC primed with the pro-inflammatory cytokine IFNγ (IFNγ-hCPC) were also used in our assay to mimic the hCPC within MI inflammatory environment.

**Figure 1:**
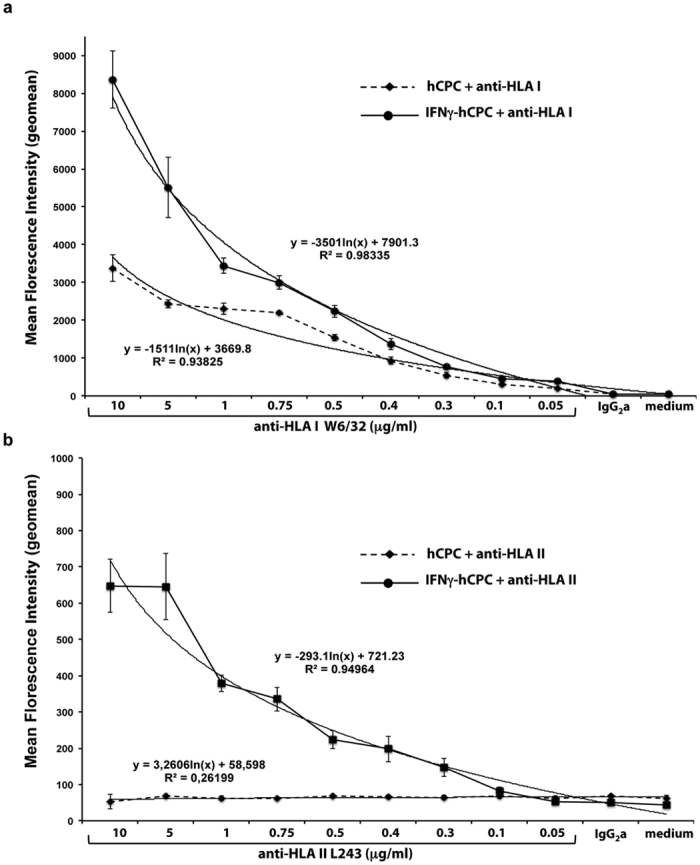
Delineation of anti-HLA mAbs interaction with hCPC by cytometry-based assay. hCPC or IFNγ-hCPC were cultured with declining concentrations of (**a**) anti-HLA-I W6/32 or (**b**) anti-HLA-II L243 mAb. The reactivity, as mean florescence intensity (MFI), for each antibody concentration was determined by flow cytometry. Results are mean MFI values ± SD obtained with hCPC (n = 6) expressing different HLA haplotypes and each tested in three different experiments. Correlation curves between MFIs and antibody concentrations for hCPC or IFNγ-hCPC along with respective R^2^ values are indicated.

The anti-HLA-I W6/32 mAb interacted with hCPC and IFNγ-hCPC in a dose-dependent manner. The highest MFI values were observed upon interaction of hCPC and IFNγ-hCPC (MFI 3000 and 8500, respectively) with the highest concentration of anti-HLA-I (10 μg/ml) and decreased thereafter displaying a logarithmic trend-line with a R^2^ value of 0.93 and 0.98, respectively (Fig. 1a). hCPC within inflammatory environment undeniably express higher levels of HLA-I antigens (Supplementary Fig. 1), which was fully reflected by the higher MFIs observed upon interaction of anti-HLA-I with IFNγ-hCPC (Fig. 1a). The anti-HLA-II L243 does not bind to hCPC at baseline since they lack the expression of HLA II. However, the anti-HLA-II interacts with IFNγ-primed HLA-II-expressing hCPC (Supplementary Fig. 1) also with logarithmic trend-line (R^2^ value of 0.94) but with an average maximal MFI value of 600 when used at 10 and 5 μg/ml (Fig. 1b). Compared to anti-HLA I, the MFIs obtained with anti-HLA-II mAb upon its interaction with IFNγ-hCPC are nearly 13 times less. Moreover, at least 0.3 μg/ml of L243 was required to observe a binding whereas a much lower concentration of anti-HLA I was sufficient (0.05 μg/ml).

Collectively, the specific recognition of the HLA-I and-II antigens on hCPC and IFNγ-hCPC by mAbs and the correlation between HLA-I and -II expression levels and the amount of anti-HLA antibodies validate the flow cytometry-based assay as a quantitative measurement that reflects both the density of HLA antigens on hCPC and the amount of the existing antibodies.

### The binding strength of anti-HLA antibodies commands hCPC susceptibility to CDC

**Figure 2:**
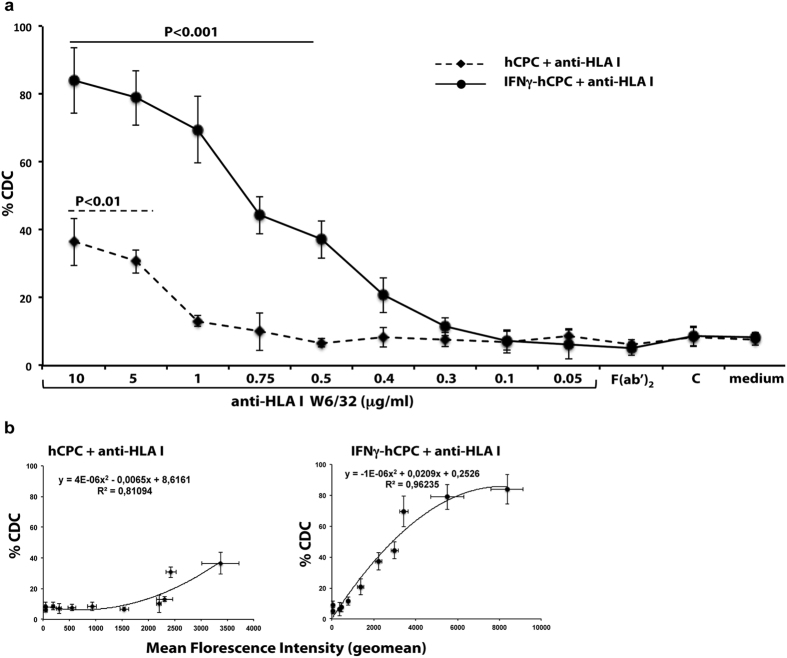
Anti-HLA antibody-induced CDC is binding strength-dependent. hCPC or IFNγ-hCPC were cultured with declining concentrations of anti-HLA-I W6/32 or 10 μg/ml of W6/32-F(ab’)_2_ with or without complement (C) then, (**a**) the capacity of anti-HLA I to induce CDC was evaluated by flow cytometry as % 7AAD-positive hCPC. (**b**) % CDC induced by each antibody concentration in hCPC (left panel) or IFNγ-hCPC (right panel) plotted as function of respective MFIs. Results are presented as mean values ± SD from three different experiments done with each hCPC. Statistical analyses were performed using One-Way Analysis of Variance (ANOVA)-Kruskal–Wallis test-dunn’s multiple comparison (GraphPadPrism Software). *P* < 0.001 and *P* < 0.01 compared to complement.

We next checked whether the binding strength of the anti-HLA mAbs as determined by the flow cytometry-based assay detected MFI could control the proneness of hCPC to antibody-mediated injury. We first checked hCPC susceptibility to CDC. hCPC and IFNγ-hCPC were cultured with medium alone or with decreasing concentrations (10 to 0.05 μg/ml) of anti-HLA-I W6/32 or anti-HLA-II L243 mAbs in the presence of complement. We then used the 7AAD dye to quantify the CDC by flow cytometry assays. The presence of 10 μg/ml of anti-HLA-I W6/32 induced the lysis of nearly 40% of hCPC. This induced CDC declined with decreasing amounts of the mAb and reached the baseline of the complement alone (Fig. 2a). The CDC was much higher in IFNγ-hCPC and nearly 90% of the cells were killed in the presence of 10 μg/ml of the W6/32 antibody. Similarly, we observed a declining lysis with decreasing amounts of the mAbs although the CDC remained significant at 0.5 μg/ml (Fig. 2a). Compared to the CDC-induced by anti-HLA-I, no lysis was observed in hCPC given their lack of HLA-II expression (Supplementary Fig. 1) and only a modest effect (around 10% specific lysis) was induced by 10 or 5 μg/ml of the anti-HLA-II L243 in IFNγ-primed HLA-II-expressing hCPC (Supplementary Figs 1 and 2). The CDC induced by different concentrations of anti-HLA-I or -II strongly correlated with their determined binding strengths with a R^2^ value of 0.81, 0.96 and 0.94, respectively (Fig. 2b and Supplementary Fig. 2). Anti-HLA-I or –HLA-II F(ab’)_2_ did not induce any significant CDC in hCPC or IFNγ-hCPC (Fig. 2a and Supplementary Fig. 2) indicating that the observed cytotoxicity is specifically induced by the ability of the complement to bind the Fc fragment of anti-HLA mAbs. These results demonstrate that anti-HLA mAbs can trigger the CDC, a major mechanism involved in acute humoral rejection, in a binding strength-depending manner.

### The binding strength of anti-HLA antibodies commands hCPC susceptibility to ADCC

Next, we analyzed the ADCC mediated through the engagement of the Fc fragment of IgG1 or IgG3 antibody by the CD16 receptor expressed by the highly cytotoxic CD56^dim^CD16^+^ NK cells. Optimum NK cell ADCC *in vitro* detection necessitates their stimulation through the shared IL2/15Rβγ receptor, in particular with IL-15^29^. Therefore, freshly-isolated NK cells were activated overnight with IL-15 (50 ng/ml) then co-cultured with allogeneic hCPC or IFNγ-hCPC in the presence or absence of different concentrations of the anti-HLA mAbs. The incidence of ADCC was then monitored by 1) the upregulation of CD137 and the expression of CD107a as readout for CD16 engagement and the degranulation of the NK cells, respectively^30^,^31^, and 2) the percentage of 7AAD-positive hCPC as readout for NK cell lytic activity.

**Figure 3:**
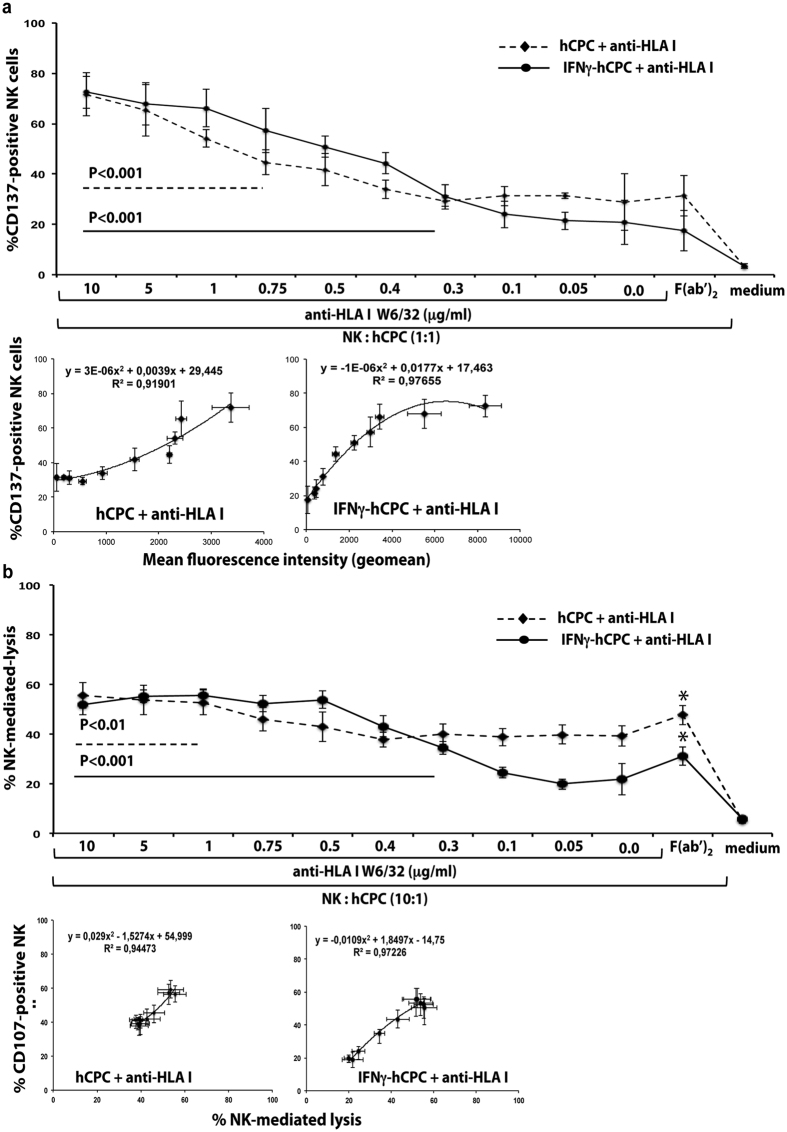
Anti-HLA antibody-induced ADCC is binding strength-dependent. IL-15-activated NK cells were cultured alone (medium) or with hCPC or IFNγ-hCPC (n = 6) in the presence of declining concentrations of anti-HLA-I W6/32 or 10 μg/ml of W6/32-F(ab’)_2_. (**a**) % CD137-positive NK cells determined by flow cytometry (upper panel). % CD137-positive NK cells observed for each antibody concentration in hCPC or IFNγ-hCPC plotted as function of respective MFIs (lower panel). (**b**) % NK cell-mediated lysis evaluated as percentage of 7AAD-positive hCPC or IFNγ-hCPC (upper panel). % CD107a-positive NK cells plotted as function of % NK-mediated lysis (lower panel). Results are presentenced as mean values ± SD from three different experiments done with each hCPC. Statistical analyses were performed using One-Way Analysis of Variance (ANOVA)-Kruskal–Wallis test-dunn’s multiple comparison (GraphPadPrism Software). *P* < 0.01 and *P* < 0.001 compared to NK + hCPC alone.

In the absence of anti-HLA-I or –HLA-II antibodies and in accordance with our previous report demonstrating that inflammatory-environment-inured hCPC are less susceptible to allogeneic NK cell killing^10^, up to 35–40% of allogeneic NK cells expressed CD137 and degranulated when co-cultured with hCPC, while only 20% of those interacting with IFNγ-hCPC (Fig. 3a upper panel, and Supplementary Figs 3 and 4). Increasing amounts of anti-HLA I W6/32 enhanced the percentage of CD137- and CD107a-positive NK cells, in the presence of hCPC, reaching a maximum of 70% and 60% at 10 μg/ml of mAb, respectively (Fig. 3a upper panel and Supplementary Fig. 3). Moreover, significant correlations were observed between the binding strength of various amounts of anti-HLA I mAb and the expression of CD137, which also significantly correlated with the degranulation of allogeneic NK cells with a R^2^ value higher than 0,91 and 0.90, respectively (Fig. 3a lower panel and Supplementary Fig. 3). The expression of CD137 and CD107a followed a similar trend when IFNγ-hCPC were used as NK cell targets (Fig. 3a lower panel and Supplementary Fig. 3) with correlation R^2^ value of 0.97.

In agreement with CD137 and CD107 expression assay, NK cells killed almost 60% of hCPC or IFNγ-hCPC in the presence of 10 to 0.5 μg/ml of anti-HLA I mAb (Fig. 3b upper panel). NK cell-mediated lysis was highly correlated with CD107a expression when hCPC and IFNγ-hCPC were used as target (R^2^ value of 0.94 and 0.97, respectively) (Fig. 3b lower panel). Compared to basal level, the anti-HLA II mAb at 10 and 5 μg/ml induced only a modest ADCC against IFNγ-hCPC. The expression of CD137 and CD107a on NK cells and the 7-ADD labeling of target cells were weakly increased (around 10%) compared to baseline (Supplementary Figs 4a,b and 5, respectively).

The presence of anti-HLA-I or -II F(ab’)_2_ (10 μg/ml) instead of full anti-HLA mAbs did not change the baseline expression of CD137 (Fig. 3a upper panel and Supplementary Fig. 4a) confirming that the observed cytotoxicity is NK cell-mediated ADCC. Of note, the baseline NK cell degranulation and lytic activity was increased in the presence of anti-HLA-I-F(ab’)_2_ (Supplementary Fig. 3 and Fig. 3b upper panel) due to the blockade of the interaction between HLA-I molecules and the NK cell inhibitory receptors KIR receptors. Such blocking would favor NK cell activation by shifting the balance between the activating and inhibitory signals that govern the NK cell cytotoxicity.

Thus, in addition to CDC the anti-HLA-I mAbs can trigger in a binding strength-depending manner the ADCC of hCPC.

### DSA-HLA-I induce CDC against hCPC

The interaction of HLA mAbs with hCPC suggests that the presence of alloantibodies, against the HLA-I or -II alleles expressed on the allogeneic donor hCPC (hereafter termed DSA-HLA-I and -II, respectively), in candidates for the cell therapy (DSA-sensitized patients) could trigger antibody-mediated injury in the therapeutic cell. We evaluated this prospect through an experimental model mimicking the clinical setting. We screened sera containing HLA panel reactive antibodies (PRA) from a cohort of heart pre-transplant patients for the presence of hCPC HLA haplotype-specific antibodies by the standard Luminex-based single-antigen flow beads technology used in transplantation. A total of 21 sera containing anti-HLA antibodies matching the HLA haplotypes (Supplementary Table 1) of the hCPC cohort (n = 6) and with Luminex-detected MFIs ranging from 1000 to 20000 were selected. Six DSA-HLA-I were directed against HLA-A2, six against HLA-A29, and three against HLA-A30 (Supplementary Table 2). For the DSA-HLA-II, we selected two against HLA-DR4, two against HLA-DR13, and two against HLA-DR1 (Supplementary Table 2).

We next determined the binding strengths of the selected 15 serum samples containing DSA-HLA I against hCPC cohort (n = 6) using our flow cytometry-based assay both at baseline and within inflammatory conditions (IFNγ-hCPC). hCPC were incubated with the DSA-HLA-I specific for their HLA-A expressed allele or with the control antibody-free serum (serum AB). FITC-conjugated anti-human Fc secondary antibody then detected the specific binding of DSA-HLA-I-A to hCPC.

**Figure 4:**
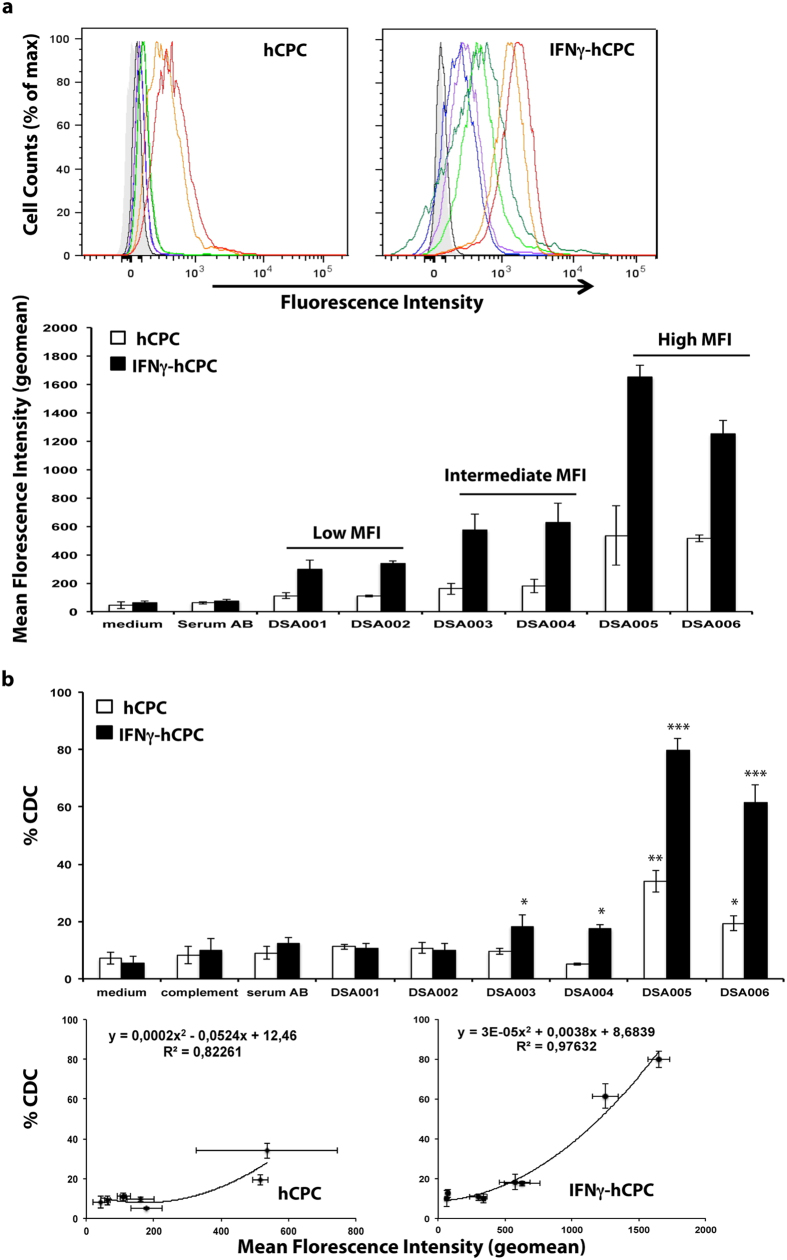
DSA-HLA-I-A2 induce CDC against hCPC. DSA-HLA-I-A2 sera (n = 6) were incubated with HLA-A2-positive hCPC or IFNγ-hCPC (n = 4), and their reactivity was determined as MFI by flow cytometry. (**a**) Upper panel showing representative histograms of DSA001 (blue), DSA002 (purple), DSA003 (light green), DSA004 (dark green), DSA005 (red), and DSA006 (orange) interactions against control serum AB (black) and medium alone (gray filled). Mean MFI (geomean) values ± SD from four different experiments of each hCPC compared to serum AB and medium controls are shown in the lower panel. (**b**) HLA-I-A2-positive hCPC or IFNγ-hCPC (n = 4) were cultured alone, or in the presence of complement with control serum AB or with DSA-HLA-I-A2 sera and their capacity to induce CDC was evaluated by flow cytometry as % 7AAD-positive hCPC (upper panel). Results are presented as mean values ± SD from four different experiments of each hCPC. The percentages of CDC induced by DSA-HLA I-A2 sera were plotted as function of respective MFIs (lower panel). Statistical analyses were performed using Mann–Whitney test for non-paired groups. *P < 0.05, **P < 0.01, ***P < 0.001 compared to hCPC in the presence of complement alone.

Regardless of their Luminex-detected MFI, all sera (n = 6) containing DSA-HLA-I against A2 (DSA001-DSA006) interacted with HLA-A2-positive hCPC (n = 4), albeit with different binding strengths (Fig. 4a upper panel). Sera were then classified as “low”, “intermediate” and “high” according to their respective cytometry-determined binding strength with hCPC or IFNγ-hCPC. DSA001 and DSA002 sera are “low” with a binding strength around 100 or 400; DSA003 and DSA004 are “intermediate” with a binding strength around 200 or 600 and DSA005 and DSA006 are “high” with a binding strength around 600 or 1600, respectively (Fig. 4a, lower panel). DSA-HLA-I against A29 (DSA007-DSA012) and A30 (DSA013-DSA015) showed similar trend of interaction with hCPC and IFNγ-hCPC expressing the HLA-A29 or HLA-A30 alleles, respectively (Supplementary Fig. 6a). Thus, DSA-HLA-I from the selected sera recognize and interact with hCPC at steady state or within inflammatory environment with a differential binding strength.

We then determined the consequences of this differential binding strength on CDC. hCPC or IFNγ-hCPC were treated with medium alone or with the complement in the absence or the presence of their respective DSA-HLA-I sera (DSA-HLA-A2, -A29, and -A30) or AB control serum, then the percentage of 7ADD-positive hCPC was assessed. Substantial CDC of hCPC and IFNγ-hCPC was mainly observed in the presence of sera containing DSA-HLA-I of high-binding strength. DSA005 and DSA006 sera containing “high” DSA-HLA-A2 induced CDC in 35% and 20% of HLA-A2-positive hCPC and in 80% and 60% of HLA-A2-positive IFNγ-hCPC, respectively (Fig. 4b upper panel). Sera containing “low” or “intermediate” DSA-HLA-A2 either did not induce any significant CDC (DSA001 and DSA002) or induced irrelevant CDC (nearly 8% compared to baseline cytotoxicity) only under inflammatory conditions. Sera containing high DSA-HLA-A29 behaved similarly (Supplementary Fig. 6b). However, the high-binding strength DSA-HLA-A30 (DSA015) induced a significant CDC only in IFNγ-hCPC probably due to the level of expression of the HLA-A30 allele on hCPC (Supplementary Fig. 6c). Compared to the AB control serum, the CDC induced by patients sera containing DSA-HLA-I, strongly correlated with their binding strength (R^2^ values > 0.90) (Fig. 4b lower panel). Sera containing alloantibodies specific for HLA-I alleles that are not expressed by the hCPC (non-DSA-HLA-I) did not induce any CDC either at steady state or under inflammatory conditions controlling the specificity of observed cytotoxicity (Supplementary Fig. 7).

Collectively, mainly the high strength binding DSA-HLA-I are able to induce significant CDC and might therefore represent a critical factor for hCPC therapy in terms of the elimination of the transplanted cells both at the steady-state and within an inflammatory environment.

### DSA-HLA-I govern NK cell cytotoxicity against hCPC

To assess the possible elimination of hCPC by NK cell-mediated ADCC mechanism in patients, we then monitored the capacity of sera with DSA-HLA-I to trigger ADCC in steady state and inflammatory environment. hCPC and IFNγ-hCPC, of HLA-A2 haplotype, were cultured with allogeneic NK cells in the presence or absence of DSA-HLA-A2 or control AB serum. The ADCC was assessed as before by monitoring both the expression of CD137 and CD107a on NK cell and the lysis of hCPC or IFNγ-hCPC.

**Figure 5:**
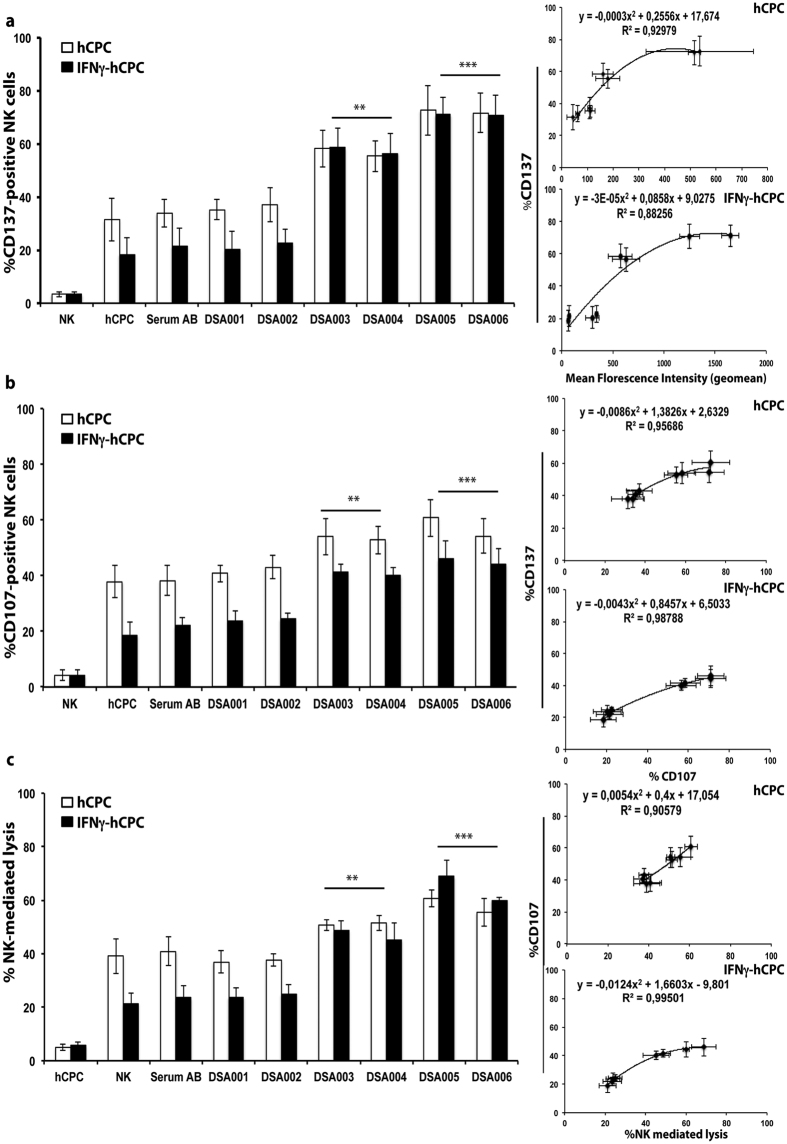
DSA-HLA I-A2 of high and intermediate binding strength induce ADCC in hCPC. IL-15-activated NK cells were cultured alone or with HLA-A2 positive hCPC or IFNγ-hCPC (n = 4) in the presence of control serum AB or DSA-HLA-I-A2 sera (DSA001-006). (**a**) % CD137-positive NK cells, (**b**) % CD107-positive NK cells, and **(C)** % NK cell-mediated lysis evaluated as % 7AAD-positive hCPC. Results represent mean values ± SD from four different experiments of each hCPC. The percentages of CD137-positive NK cells with hCPC or IFNγ-hCPC were plotted as function of respective MFIs of DSA-HLA-I-A2 sera (upper right panel) or as function of % CD107-positive NK cells (middle right panel), the % CD107-positive NK cells were plotted as function of % NK-mediated lysis (low right panel) for both hCPC and IFNγ-hCPC. Statistical analyses were performed using Mann–Whitney test for non-paired groups. **P < 0.01 and ***P < 0.001 compared to NK + hCPC.

The presence of DSA-HLA-A2 sera of high binding strength increased the percentage of baseline CD137^+^ NK cells by more than 2 folds compared to control co-cultures (Fig. 5a). The CD137^+^ over expression on NK cells was associated with an increase in NK cell degranulation (Fig. 5b) and cytotoxic activity (Fig. 5c). In contrast to CDC experiments, intermediate strength DSA-HLA-A2 (DSA003 and DSA004) sera were able to mediate significant ADCC in both hCPC and IFNγ-hCPC increasing by 1.2–2 folds the baseline allogeneic NK cell cytotoxicity (Fig. 5c). Similar increase of the allogeneic NK cell cytotoxicity against HLA-I-A29 and -A30-positive hCPC or IFNγ-hCPC was also observed in the presence of high and intermediate binding strength DSA-HLA-A29 and DSA-HLA-A30 sera, respectively (Supplementary Figs 8 and 9). The low binding strength DSA-HLA-I, regardless of their targeted HLA-I specificity, did not incite any significant ADCC against hCPC or IFNγ-hCPC (Fig. 5, and Supplementary Figs 8 and 9).

Again, compared to the AB control serum, the ADCC induced by sera containing DSA-HLA I, strongly correlated with their binding strength. Significant correlations were observed between the binding strength of DSA-HLA I containing sera to hCPC and the expression of CD137, which also significantly correlated with the degranulation and cytotoxicity of allogeneic NK cells (R^2^ values higher than 0.92, 0.95, and 0.90, respectively) (Fig. 5, and Supplementary Figs 8 and 9). The expression of CD137 and CD107a as well as the NK cell cytotoxicity followed a similar trend when IFNγ-hCPC were used as NK cell targets (Fig. 5, and Supplementary Figs 8 and 9) with correlation R^2^ values higher than 0.88, 0.95, and 0.99, respectively.

**Figure 6:**
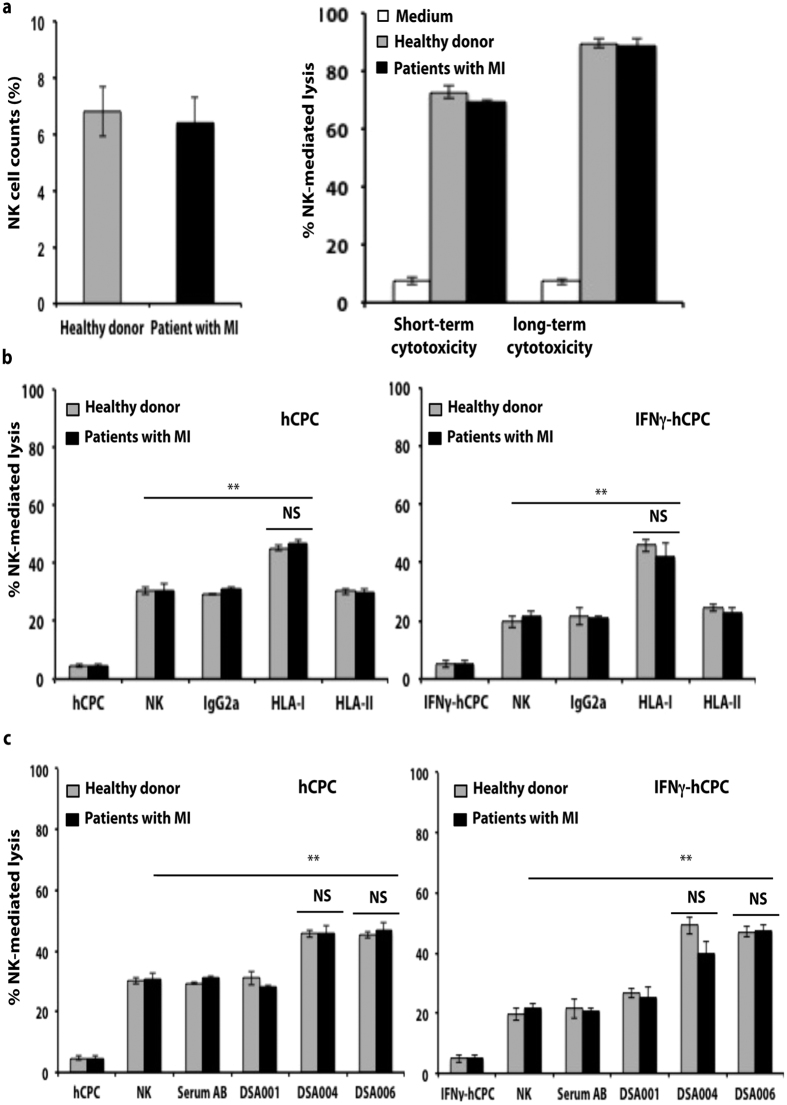
NK cells from patients with MI behave similar to NK cells from healthy donors. IL-15-activated NK cells from healthy donors (n = 3) or from patients with MI (n = 3) were cultured alone or with HLA-A2-positive hCPC or -IFNγ-hCPC (n = 2) in the presence of control serum AB or DSA-HLA-I-A2 sera (DSA001, DSA004, DSA006). **(a)** Percentages of NK cells present in total PBMC (left panel) and % NK cell-mediated lysis in NK-target k562 cells evaluated as % 7AAD-positive k562 (right panel). (**b**) % NK cell-mediated lysis evaluated as % 7AAD-positive hCPC (right panel) or IFNγ-hCPC (left panel) in the presence of mAb and (**c**) in the presence of DSA-HLA I. Results represent mean values ± SD from 3 different experiments. Statistical analyses were performed using Mann–Whitney test for non-paired groups. NS: non-significant **P < 0.01compared to NK + hCPC.

Patients with heart diseases might display reduced number of cytotoxic CD16^+^ CD56^dim^ NK cells and a concomitant reduced lytic activity^32^. To understand whether NK cell effector function was impaired in MI patients, we isolated NK cells from PBMC of three MI patients and analyzed their function. Compared to healthy donors, patients with MI showed similar percentages of NK cells (Fig. 6a left panel). Moreover, NK cells from MI patients and healthy donors showed similar cytotoxicity against the NK cell target cells K562 (Fig. 6a, right panel). MI patient’s NK cells are also able to induce ADCC in hCPC and IFNγ-hCPC only in the presence of mAb anti-HLA I W6/32 (10 μg/ml) (Fig. 6b). Similar to healthy donors, NK cells from MI patients are able to mediate ADCC in the presence of intermediate and high strength DSA-HLA-A2 (DSA004 and DSA006, respectively) but not in the presence of the low strength DSA001. We observed 1.5 to 2-folds increases of baseline NK cell cytotoxicity towards allogeneic hCPC and IFNγ-hCPC respectively (Fig. 6c).

Together, our data demonstrate that the capacity of DSA-HLA-I to activate NK cell ADCC against hCPC is correlated to their binding strength. The DSA-HLA-I-sensitization alters the NK cell response against allogeneic hCPC whereby its transition from modest-favorable to deleterious might accelerate the clearance of the implanted cells.

### DSA-HLA-II recognize IFNγ-hCPC but do not trigger CDC or ADCC

We did not observe any relevant CDC or ADCC against hCPC with anti-HLA-II mAb, however we analyzed whether DSA-HLA-II can trigger either CDC or NK cell ADCC mechanisms. The six DSA-HLA II sera (Supplementary Table 2) specific for the HLA-DR haplotypes expressed by the hCPC cohort (n = 6) were evaluated.

**Figure 7:**
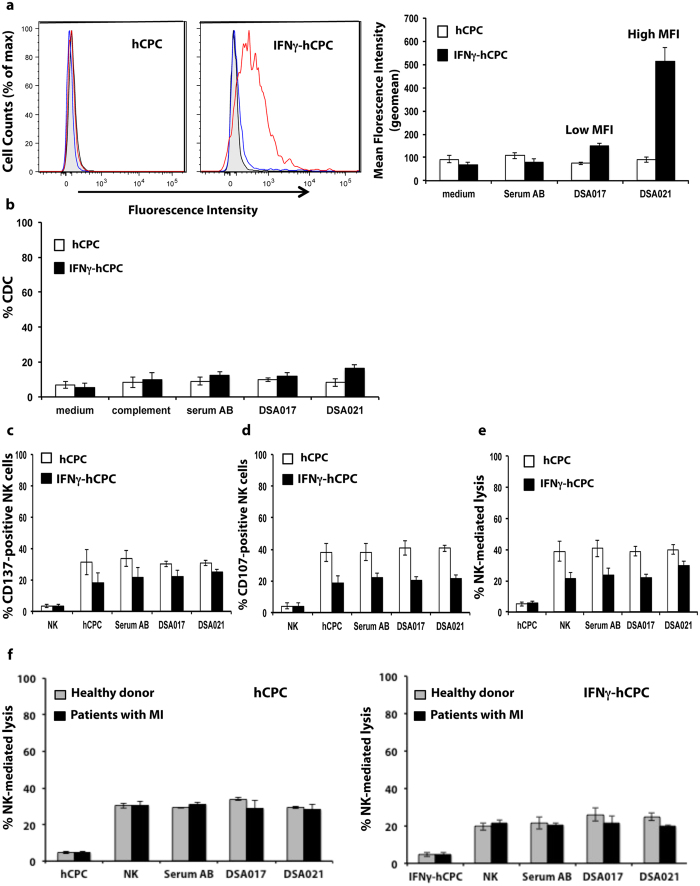
DSA-HLA-II do not induce antibody-mediated cytotoxicity. DSA-HLA-II-DR4 sera (n = 2) were incubated with HLA-DR4-positive hCPC or IFNγ-hCPC (n = 2) then their reactivity was determined as MFI by flow cytometry. (**a**) Left panel showing representative histograms of DSA017 (blue) and DSA021 (red) interactions against control serum AB (black) and medium alone (filled gray). Mean MFI (geomean) values ± SD from three different experiments of each hCPC compared to serum AB and medium controls (right panel). (**b**) HLA-DR4-positive hCPC or IFNγ-hCPC (n = 2) were cultured alone, with control serum AB, or with DSA-HLA-II-DR4 sera (DSA017, 021), in the presence or absence of complement, then their capacity to induce CDC was evaluated as % 7AAD-positive hCPC. Results are presented as mean values ± SD from four different experiments of each hCPC. (**c–e**) IL-15-activated NK cells were cultured alone or with HLA-DR4-positive hCPC or IFNγ-hCPC (n = 2) in the presence of control serum AB or DSA-HLA-II-DR4 sera. (**c**) % CD137-positive NK cells, (**d**) % CD107-positive NK cells and (**e**) % NK-mediated lysis evaluated as % 7AAD-positive hCPC. Results represent mean values ± SD from three different experiments of each hCPC. (**f**) % Healthy- or patient-NK-mediated lysis evaluated as % 7AAD-positive hCPC. Results represent mean values ± SD from three different experiments. Statistical analyses were performed using Mann–Whitney test for non-paired groups and were non-significant.

None of the DSA-HLA-II sera interacted with hCPC given their lack of HLA-II expression. Under inflammatory conditions, different binding strengths were observed for the different sera (Fig. 7a, and Supplementary Figs 10a and 11a). Similar to DSA-HLA-I, we categorized the DSA-HLA-II as “low”, “intermediate” or “high” according to their binding strength to IFNγ-hCPC. DSA-HLA-II-DR4 (DSA017), DSA-HLA-II-DR13 (DSA018), and DSA-HLA-II-DR1 (DSA016) sera with a binding strength around 150 were considered “low”; DSA-HLA-II-DR13 (DSA020), and DSA-HLA-II-DR1 (DSA019) sera with a binding strength around 300 were considered “intermediate” and DSA-HLA-II-DR4 (DSA021) serum with the highest binding strengths (600) was considered “high”. None of the analyzed DSA-HLA-II or non-DSA-HLA-II was able to trigger relevant CDC (Fig. 7b, and Supplementary Figs 10b, 11b, and 7) or ADCC (Fig. 7c–e, and Supplementary Figs 10c–e, 11c–e). Similar results were obtained with NK cells from patients with MI (Fig. 7f).

Thus, although the DSA-HLA-II are able to interact with inflammatory-environment-inured hCPC, they are unable to elicit any antibody-mediated cytotoxicity in these cells regardless of their specific haplotype or binding strength.

### Luminex-based anti-HLA antibody screening towards more efficient hCPC-based therapy

In clinical allogeneic transplantations, sensitive solid-phase assays using Luminex-based technology are the standard practice to detect the presence and identify the specificities of DSA-HLA^18^. They establish a correlation between the *in vitro* antibody reaction, measured as MFI representing the amount of antibody bound relative to the total antigen present on the beads, and the eventual clinical outcome in terms of rejection or engraftment. However, this approach was never addressed in the context of the hCPC-based therapy. Therefore, we explored its suitability to eventually guide the choice of hCPC therapeutic cells.

**Figure 8:**
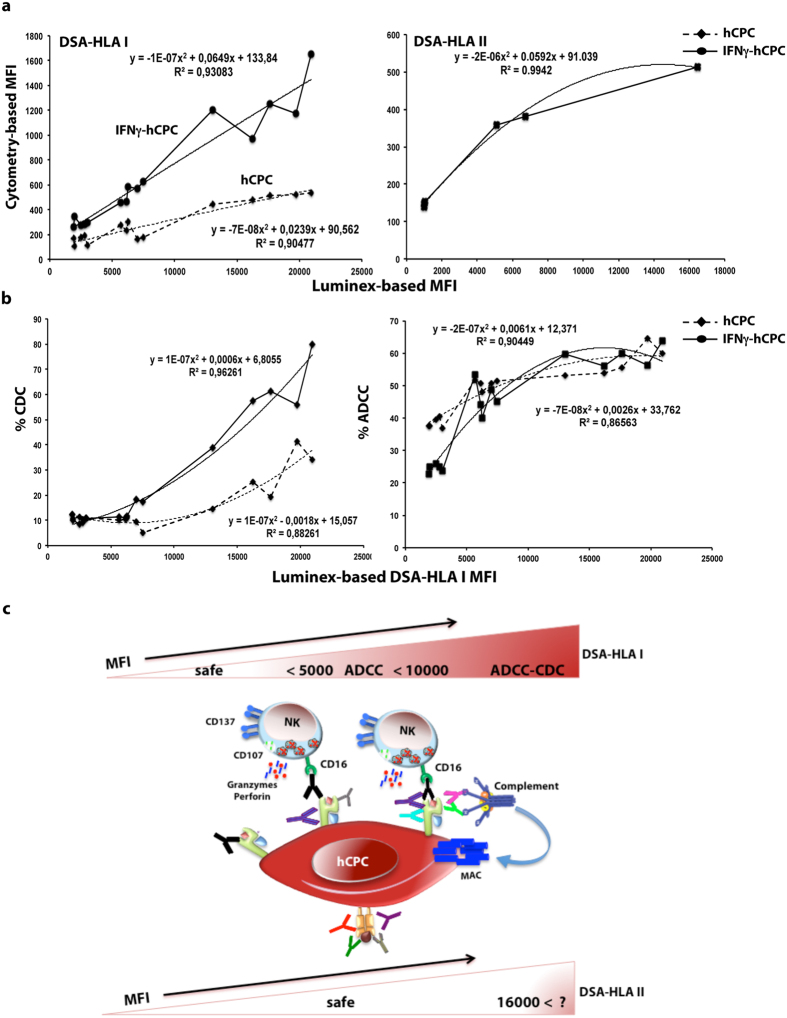
Translational dimension of DSA-HLA sensitization in the context of hCPC therapy. (**a**) Left panel DSA-HLA-I (n = 15) and right panel DSA-HLA-II (n = 6) sera flow cytometry-detected MFIs values were plotted as function of their Luminex-detected MFIs. (**b**) Left panel % CDC and right panel % ADCC against hCPC or IFNγ-hCPC plotted as function of their Luminex-detected MFIs. Correlations curves along with their respective R^2^ values are indicated. (**c**) Schematic representation of DSA-HLA-sensitization risk for hCPC-based therapy. DSA-HLA-I of Luminex-detected high mean florescence intensity (MFI) present an absolute risk of humoral rejection inducing both CDC and ADCC through complement activation and the formation of the membrane attack complex (MAC) or by bridging NK cells CD16 receptor with HLA-I molecules at the surface of hCPC, respectively. DSA-HLA-I with Luminex-detected MFIs ranging from 5000 to 10000 present a relative risk of humoral rejection bridging NK cells CD16 receptor with HLA-I molecules at the surface of hCPC will induce only ADCC. DSA-HLA-I having Luminex-detected MFIs < 5000 and DSA-HLA-II with MFI up to 16000 are safe.

Regardless of their HLA-I antigen specificity, we found that the DSA-HLA-I classified as “low” by flow cytometry-based assay had an MFI < 4000 by Luminex-based assay, those classified as “intermediate” had an MFI higher than 4000 but less than 10000 by Luminex-based assay, and those classified as “high” showed an MFI > 10000 by Luminex-based assay. Moreover, the MFI determined by the flow cytometry- and the Luminex-based assay followed a polynomial curve with significant R^2^ values of 0.90 and 0.93 for hCPC at baseline and IFNγ-hCPC, respectively (Fig. 8a left panel). The DSA-HLA-II cytometry-detected MFIs, when plotted as function of DSA-HLA-II Luminex-detected MFIs also followed a polynomial curve with a significant R^2^ value of 0.99 (Fig. 8a right panel). Although of much lower values, the DSA-HLA-I MFI determined by flow cytometry mirrored those determined by Luminex-based assay. A significant correlation was also obtained between DSA-HLA I Luminex-based MFIs and the occurrence of CDC or ADCC in hCPC (Fig. 8b). Collectively, these significant correlations would prompt the suitability of Luminex-based DSA-HLA screening both in pre- and post-infusion of hCPC to predict the likelihood of their immune-mediated loss.

## Discussion

Lessons from organ and HSC transplantation indicate that optimizing the prediction and control of deleterious cellular and humoral immune risk factors is decisive for the clinical outcome of allogeneic therapies. In addition to off-the-shelf availability, allogeneic hCPC-based therapy has become a prominent strategy for cardiac repair because of their favorable allogeneic cellular immune consequences that have been linked, in part or as whole, to their beneficial effects^7^,^8^,^10^. Herein, by using an hCPC-tailored flow cytometry-based assay, we demonstrate that allogeneic hCPC are prone to DSA-HLA-I-induced injury but seem rather invulnerable to DSA-HLA-II. The occurrence of CDC and ADCC is governed by the cytometry-detected binding strength of the HLA antibodies within the experimental settings. Thus, screening patients for HLA antibody would estimate the risk of DSA-HLA sensitization and immune educate the choice of allogeneic hCPC towards beneficial balanced allogenecity.

In allogeneic transplantations, depending on antigen density on the target and capacities of the antibody Fc-domain, cells and tissues have different susceptibility for damage by antibodies^18^. Both the density of HLA antigens on hCPC and the amount/capacity of patient hCPC-specific anti-HLA antibodies also orchestrate the occurrence of antibody-dependent cell death mechanisms. At baseline, hCPC express considerable levels of HLA-I but lack HLA-II antigens. When stimulated by IFN-γ, that mimic post-MI inflammatory environment, hCPC express HLA-II antigens albeit at a much lower density than HLA-I antigens. The signal/concentration curves obtained for HLA-I and HLA-II mAbs were different at baseline and within inflammatory environment suggesting that the signal intensity depends on the density of antigens present at the cell surface of hCPC. The fact that both the positivity and saturation thresholds of cytometry-based MFI for anti-HLA-I mAb were much higher than those for anti-HLA-II mAb within inflammatory environment, further support the density of antigens as a key determinant of signal intensity.

Plotting the percentage of CDC triggered by different concentrations of monoclonal HLA antibodies as a function of fluorescence values (MFI) followed a non-linear polynomial curve with significant R^2^ values. The mouse IgG2a Fc-domain of these HLA mAbs is analogue to the human IgG1 that can effectively activate the complement^33^,^34^,^35^. Therefore, the CDC/MFI curve models a functional relationship between these values and establishes a significant correlation between cytometry-based *in vitro* antibody reactions measured by MFI and possible fate of hCPC infusion in terms of rejection or engraftment.

In addition to the CDC pathway, the NK cell-mediated ADCC is another risk factor for chronic cardiac graft rejection^21^,^36^,^37^. NK cell cytotoxic function occurs through natural cytotoxicity or ADCC^38^. We have previously reported that the inflammatory environment significantly decreases the susceptibility of allogeneic hCPC to NK cell natural cytotoxicity and promotes anti-inflammatory cytokines secretion through mechanisms involving HLA-I antigens and NK cell inhibitory receptors (KIR)^10^,^38^. Here, we show that the binding of HLA-I mAb, but not HLA-II mAb, to hCPC triggers significant NK cell ADCC trough the ligation of the mAb Fc fragment to its FcγRIIIa NK cell receptor (CD16). Furthermore, low concentration of HLA-I mAb was sufficient to activate NK cells and induce significant killing of IFNγ-hCPC. Accordingly, it is very unlikely that the post-MI inflammatory environment would protect allogeneic hCPC from NK cell-mediated ADCC in the presence of HLA I antibodies.

The presence of anti-HLA-I F(ab’)_2_ in co-cultures of both hCPC and IFNγ-hCPC resulted in higher NK cell natural cytotoxicity compared to control baseline (co-culture NK/hCPC). This might be due to decreased interaction of NK cells inhibitory KIRs with their HLA-I ligands^39^. Only high concentrations of anti-HLA-II antibody were able to modestly increase NK-mediated killing of IFNγ-hCPC. Since CD16 have similar affinities to Fc-domains of both HLA-I and HLA-II mAbs^33^,^34^,^35^, the modest HLA-II mAb-induced ADCC is probably due to low antigen density on IFNγ-hCPC.

Other innate immune cells including macrophages express different sub-classes of Fcγ receptors and might mediate ADCC and graft rejection^40^,^41^. For instance, in allogeneic hepatocytes and islet cell transplantation models, the presence of alloantibodies resulted in macrophage-mediated ADCC and graft rejection^42^,^43^. In addition, the presence of alloantibodies enhances monocytes trafficking and macrophages accumulation in solid organ allografts and might lead to their damage^44^,^45^. In experimental MI, injection of allogeneic cardiosphere-derived cells (CDCs) in experimental MI models confers cardio-protection and limits injury and inflammation through rapid reduction of pro-inflammatory effector macrophages and induction of protective phenotype^46^. Whether the presence of alloantibodies would influence such rapid macrophage-mediated protection/tissue repair remains as yet an open question. Studies examining the effects of allogeneic cardiac-derived stem/progenitor cells on monocytes/macrophages in the presence or absence of alloantibodies are therefore, warranted to clarify this risk.

The reactivity of allosera from a cohort of heart pre-transplant patients containing anti-HLA-I or –HLA-II alloantibodies that match our hCPC haplotypes suggests a detrimental role of the DSA-HLA-I positivity, which might however vary in function of HLA-I allele. In fact, allosera containing DSA-HLA-A30 with cytometry-detected MFI of 440 induced significant CDC against hCPC only in inflammatory environment. In contrast, allosera containing DSA-HLA-A2 or -A29 with slightly higher MFIs induced significant CDC in both inflammatory and steady state conditions. In healthy individuals, the expression levels of HLA-I and -II are allele dependent. This differential expression of HLA antigens is of great relevance in clinical setting^47^,^48^,^49^,^50^. Thus, beside DSA-HLA-I binding strength, their risk to hCPC should probably be viewed in the context of the HLA alleles of therapeutic cells.

Within the clinical setting, the screening for the DSA-HLA and the cross-match testing using flow cytometry-based assay would be difficult to perform. In fact, the high technical strains inherent to stem cell manipulations would incite the use of the standard bead-based multiplex Luminex assays. Prediction would then be by comparing the potential patient’s HLA-specific antibodies with the HLA type (allelic lineage) of therapeutic hCPC. We found that significant correlations between the flow cytometry-detected and Luminex-detected MFIs. Luminex-detected MFIs also significantly correlated with occurrence of CDC and ADCC in hCPC providing thus, a translational dimension to our flow cytometry-based screening and cross-match data.

The fact that allosera containing DSA-HLA-II (DSA-HLA-DR) with high Luminex-detected binding strength induced neither CDC nor ADCC in inflammatory-environment-inured hCPC is most likely due to the low expression of HLA-DR antigens. This does not exclude the possibility that DSA-HLA-DR with much higher MFI or with other specificities (other alleles) than those studied in this report might react with hCPC at higher intensity and provoke antibody-mediated deleterious effects. This might be supported by our results showing that interaction of DSA-HLA II with inflammatory-inured hCPC follows a polynomial curve with significant R^2^ values of 0.99.

Beside HLA-DR isoform, inflammatory-environment-inured hCPC would probably express low levels of the other HLA-II isotypes namely HLA-DQ and HLA-DP (Supplementary Fig. 1). Antibodies against these two isotypes are also frequent among HLA II-sensitized patients, and their clinical relevance in terms of chronic rejection and engraftment allografts has been demonstrated in retrospective studies^51^,^52^. Since allogeneic hCPC express low levels of HLA-DQ and HLA-DP antigens under inflammatory conditions, it very unlikely that DSA-HLA-DQ or DSA-HLA-DP alone would be harmful. Yet, we cannot fully roll out a synergistic effect between DSA-HLA-DR and DSA-HLA-DQ and/or DSA-HLA-DP.

Alongside CDC and ADCC classical antibody-induced mechanisms, the relevance of antibody-induced HLA signaling in transplantation has been demonstrated^13^,^53^. While HLA molecules are principally viewed as antigen presenting structures they are also bona fide signal transduction receptors^54^. Therefore, HLA-mediated signaling after ligation of HLA antigens on hCPC with specific alloantibodies is conceivable and might also contribute to the regulation of their fate. Here again, the level of alloantibodies would probably govern the outcome of HLA signaling. Saturating concentrations of HLA-I antibodies and complement induces death of endothelial cells while low doses promote their survival and confer resistance to CDC^55^. In addition, HLA signaling may synergize with Fc-dependent effector functions, including activation and binding to Fcγ receptors on monocytes/macrophages, and promote an enhanced state of inflammation in the allografts^56^. In this report, the exposure of hCPC to low doses of HLA-I mAb, low-MFI DSA-HLA-I, to anti-HLA-DR mAb or DSA-HLA-II does not induce any significant classical antibody-mediated injury. Whether through HLA signaling such exposure might promote injury/inflammation or rather survival conferring resistance to hCPC is as yet unexplored and warrant investigations.

Allogenicity is a striking example of a system that can produce both beneficial as well as detrimental effects, raising important conceptual, experimental and clinical issues. In regenerative/reparative medicine, stem/progenitor cells should persist long enough either to differentiate or to activate endogenous regeneration/repair. We have demonstrated that allogeneic hCPC have benefic cellular consequences. These cells incite immunomodulatory anti-inflammatory response and contribute to injured myocardium repaire^8^,^10^. This study demonstrates the potential humoral risk of hCPC allogenicity (Fig. 8c), which might trigger their pre-mature elimination upon infusion and limit their beneficial effects. Thus, the allogenicity of hCPC is a “Yin-Yang” opposing forces forming a dynamic system. Therefore, minimizing the risk while optimizing the benefit is an important notion for an ultimate efficient allogeneic hCPC-based therapy. The question is what is the acceptable mismatching and how much risk could be allowed in regard of these fast-track therapies. This would be even more important to avoid the immunization that would be detrimental if recipient of allogeneic hCPC therapy become later eligible for heart transplantation or if repeated administration of allogeneic cells is needed. By analogy to allogeneic organ and tissue transplantations, the “type-and-screen” strategy to HLA-match and reduce HLA-mismatch would be an ideal approach for allogeneic hCPC therapy.

In summary, a systematic immuno-monitoring of DSA-HLA by Luminex-based assay would provide an immune-educated choice of off-the-shelf allogeneic hCPC that might extend their persistence to activate the endogenous regeneration and/or repair of the impaired heart function. Our study advocates immune-educated choice of allogeneic therapeutic cardiac-derived stem/progenitor as a new approach towards a better clinical success.

## Methods

An expanded Materials and Methods section is available in the on- line-only.

### Study design

Human cardiac biopsies were obtained from patients undergoing open-chest surgery after signed informed consent in accordance with the Declaration of Helsinki. The ethical committees of “Hospital 12 de Octubre”, “Fundación Jiménez Díaz”, (Madrid) and “Complejo Hospitalario de Navarra” (Pamplona) - Spain have approved the project. Cryopreserved cardiac stem/progenitor cells (hCPC) were obtained from the right atria appendage after immunodepletion of CD45-positive cells and immunoselection of CD117 (c-kit) at Cortherapix (currently, Tigenix) as descried^8^. The hCPC (n = 6) from different donors were genotyped for HLA using standard techniques (Supplementary Table 1). hCPC-tailored flow cytometry-based assays were established to assess the capacity of HLA antibodies to interact with hCPC and determined the characteristic of this interaction. Cryopreserved allosera from heart transplantation patients (pre-transplantation) containing panel reactive antibodies (PRA) – allosera containing HLA antibodies, were provided by the Laboratory of Immunology and Histocompatibility, Saint Louis Hospital, Paris, France. Allosera were obtained in accordance with the local institutional regulations and the approval of the local ethic committee, the “Comité consultatif pour la protection des personnes dans les recherches biomédicales”, and used as a model to mimic the clinical setting. All patients provided informed consent allowing for data submission to Laboratory of Immunology and Histocompatibility database at the Saint Louis Hospital and use of data for research in accordance with the Declaration of Helsinki. Luminex-based single-antigen flow bead technology was used to screen serum samples for the presence of anti-HLA antibodies matching the HLA haplotypes of the hCPC cohort (n = 6). A total of 21 sera containing hCPC-specific DSA-HLA (DSA-HLA-I against HLA-A and DSA-HLA-II against HLA-DR) and displaying Luminex-based binding strength greater than 500 (Supplementary Table 2) were selected and used to assess the susceptibility of hCPC to CDC and ADCC. Experiments were performed at 3% O_2_ with passages 3 and 7 hCPC and IFNγ-hCPC, mimicking those primed/stimulated by inflammatory environment, at 80–90% confluence and reproduced in at least three independent experiments with each hCPC as stated in the figure legends. IFNγ-hCPC were washed before co-culture with NK cells to avoid any stimulation of the immune cells by residual IFN-γ. Studies of correlation were performed to demonstrate the humoral risk of allogeneic hCPC and the translational relevance of our findings for hCPC-based therapy.

The study was approved by the local research ethical comity, the “Comité consultatif pour la protection des personnes dans les recherches biomédicales”. All experiments were performed in accordance with the local institutional guidelines and regulations and the approval of the local ethic committee, and with informed consent from all subjects.

### Natural Killer (NK) cells purification

Peripheral blood mononuclear cells (PBMC) were prepared from blood samples of healthy donors (n = 10) as well as from patients with MI (n = 3) and genotyped for HLA using standard techniques. A signed informed consent following human ethics committee “Comité consultatif pour la protection des personnes dans les recherches biomédicales” - Saint Louis Hospital, Paris, France) has been obtained from all the donors, and all methods and experimental protocols were approved by the by the institution and were conducted in accordance with guidelines and regulation. All experiments implicating NK cells were conducted in allogeneic settings with cells activated overnight with recombinant human IL15 (50 ng/mL) (Immunotools, Friesoythe, Germany).

### Immune phenotyping

The expression level of HLA-I and -II on hCPC and IFNγ-hCPC was determined by flow cytometry using specific anti-HLA mAbs and PE-conjugated goat anti-mouse IgG secondary antibody (Online Table 3).

### Complement-Dependent-Cytotoxicity – hCPC-tailored cross-match

hCPC or IFNγ-treated hCPC were incubated with anti-HLA-I or anti-HLA-II mAbs at different concentrations or with allosera. Pure complement was then added and 7AAD staining assessed the % of lysed hCPC.

### Antibody-dependent cell-mediated cytotoxicity

Freshly-isolated IL-15-activated NK cells were co-cultured with hCPC or IFNγ-hCPC in the presence or the absence of different concentrations of anti-HLA mAbs or allosera. NK degranulation was determined by anti-CD107a mAb staining whereas engagement of CD16 was determined by anti-CD137 staining. Cells were acquired on Canto II flow cytometer and NK cells were gated as CD3^−^CD56^+^ cells and analyzed by BD FACS Diva software. To determine NK cell-mediated hCPC lysis, **c**arboxyfluorescein succinimidyl ester (CFSE)-labeled hCPC or IFNγ-hCPC were co-cultured with IL-15-activated NK cells in the presence or absence of various concentrations of mAbs and % of CFSE-labeled 7-AAD-positive-hCPC or -IFNγ-hCPC was determined.

### Statistical analysis

Statistical analyses were performed using Mann–Whitney test for non-paired groups, paired Student’s t-test for paired groups and One-Way Analysis of Variance (ANOVA)-Kruskal–Wallis test-dunn’s multiple comparison for multiple comparison (GraphPadPrism Software). Data are expressed as mean value ± SD, P-values < 0.05 were considered significant. *P < 0.05, **P < 0.01, ***P < 0.001.

## Additional Information

**How to cite this article:** Hocine, H. R. *et al*. Minimizing the risk of allo-sensitization to optimize the benefit of allogeneic cardiac-derived stem/progenitor cells. *Sci. Rep.* **7**, 41125; doi: 10.1038/srep41125 (2017).

**Publisher's note:** Springer Nature remains neutral with regard to jurisdictional claims in published maps and institutional affiliations.

## Supplementary Material

Supplementary Information
