# Corrigendum: Minimizing the risk of allo-sensitization to optimize the benefit of allogeneic cardiac-derived stem/progenitor cells

**DOI:** 10.1038/srep44720

**Published:** 2017-03-16

**Authors:** Hocine R. Hocine, Hicham E. L. Costa, Noemie Dam, Jerome Giustiniani, Itziar Palacios, Pascale Loiseau, Armand Bensussan, Luis R. Borlado, Dominique Charron, Caroline Suberbielle, Nabila Jabrane-Ferrat, Reem Al-Daccak

Scientific Reports 7: Article number: 4112510.1038/srep41125; published online: 11 24 2017; updated: 03 16 2017

The original version of this Article contained a typographical error in the spelling of the author Armand Bensussan which was incorrectly given as Armand Benssusan. This has now been corrected in the PDF and HTML versions of the Article.

