# Corrigendum: Minimizing the risk of allo-sensitization to optimize the benefit of allogeneic cardiac-derived stem/progenitor cells

**DOI:** 10.1038/srep46888

**Published:** 2017-10-20

**Authors:** Hocine R. Hocine, Hicham El Costa, Noemie Dam, Jerome Giustiniani, Itziar Palacios, Pascale Loiseau, Armand Bensussan, Luis R. Borlado, Dominique Charron, Caroline Suberbielle, Nabila Jabrane-Ferrat, Reem Al-Daccak

Scientific Reports 7: Article number: 41125; 10.1038/srep41125 published online: 01 24 2017; updated: 10 20 2017.

The original version of this Article as well as the previous Corrigendum contained a typographical error in the spelling of the author Hicham El Costa which was incorrectly given as Hicham E.L. Costa.

This error has now been corrected in the PDF and HTML versions of the Article, as well as the previous Corrigendum that accompanies the Article.

