# Supplementary material for: Minimizing the risk of allo-sensitization to optimize the benefit of allogeneic cardiac-derived stem/progenitor cells
Source: Sci Rep. 2017 Jan 24;7:41125. doi: 10.1038/srep41125 (PMC5259698; doi:10.1038/srep41125)
Supplement: Supplementary Information [file srep41125-s1.pdf]

SUPPLEMENTARY MATERIAL

Minimizing the risk of allo-sensitization to optimize the benefit of allogeneic cardiac-derived stem/progenitor cells

Hocine R. Hocine[1,2], Hicham EL Costa[3], Noemie Dam[1,4], Jerome Giustiniani[1], Itziar Palacios[4], Pascale Loiseau[5], Armand Benssusan[1], Luis R. Borlado[4], Dominique Charron[1,2], Caroline Suberbielle[2,5], Nabila Jabrane-Ferrat[3*], and Reem Al-Daccak[1,2*]

*Corresponding author: reem.al-daccak@inserm.fr

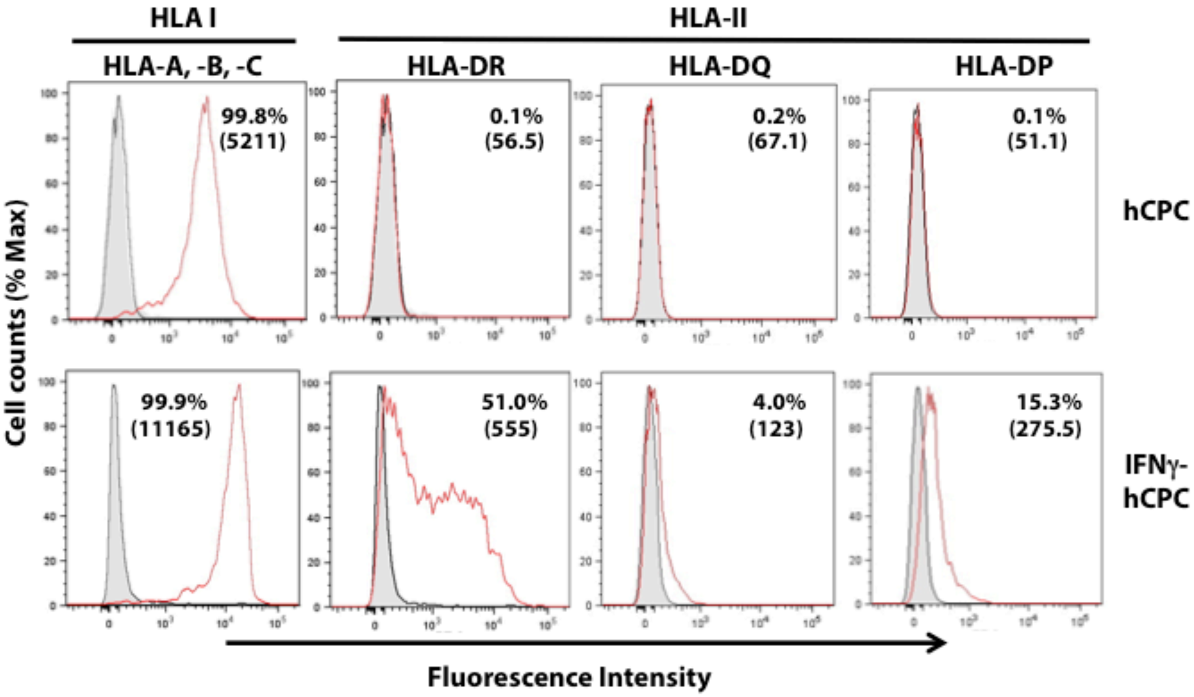

Supplementary Figure 1

Expression of HLA-I and -II antigens on hCPC and IFNγ-hCPC. Representative histograms of surface expression of HLA-I and -II antigens on hCPC and IFNγ-treated (500IU/ml) hCPC (red) against respective isotype controls (black) and medium (filled gray). Percentage of positive cells and geometric means () are indicated.

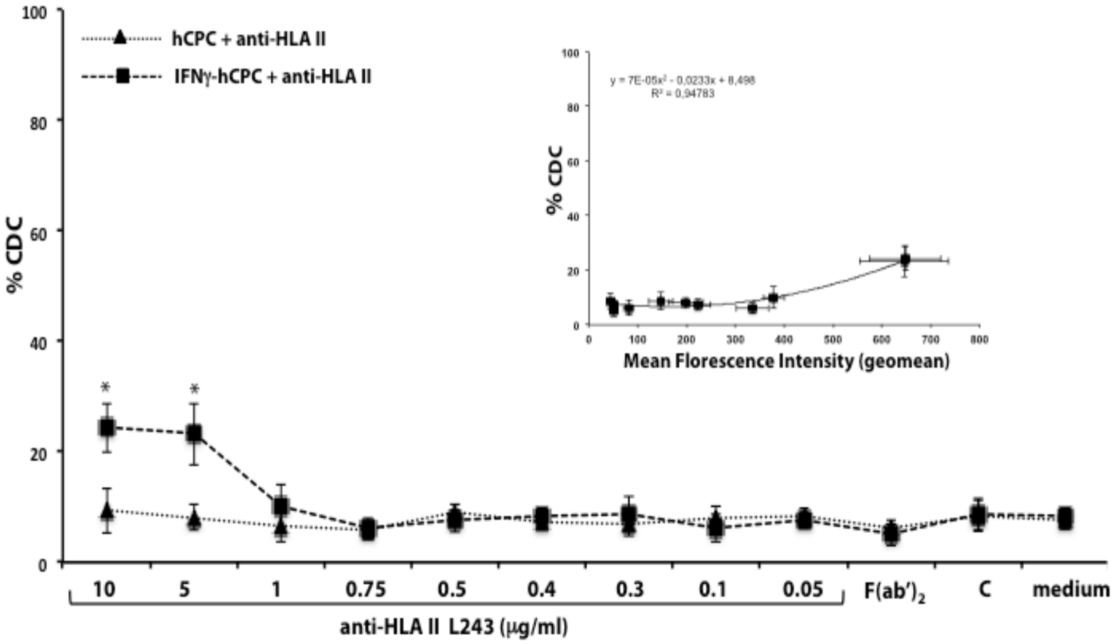

Supplementary Figure 2

Anti-HLA-II induced CDC. hCPC or IFNγ-hCPC were cultured with declining concentrations of anti-HLA-II L243 or 10μg/ml of L243-F(ab')$_2$ with or without complement (C), then the capacity of anti-HLA II to induce CDC was evaluated by flow cytometry as % 7AAD-positive hCPC. % CDC induced by each antibody concentration IFNγ-hCPC plotted as function of respective MFIs (imbedded curves). Results are presented as mean values±SD from three different experiments done with each hCPC. Statistical analyses were performed using One-Way Analysis of Variance (ANOVA)-Kruskal–Wallis test-dunn's multiple comparison (GraphPadPrism Software). *P<0.05 compared to complement alone.

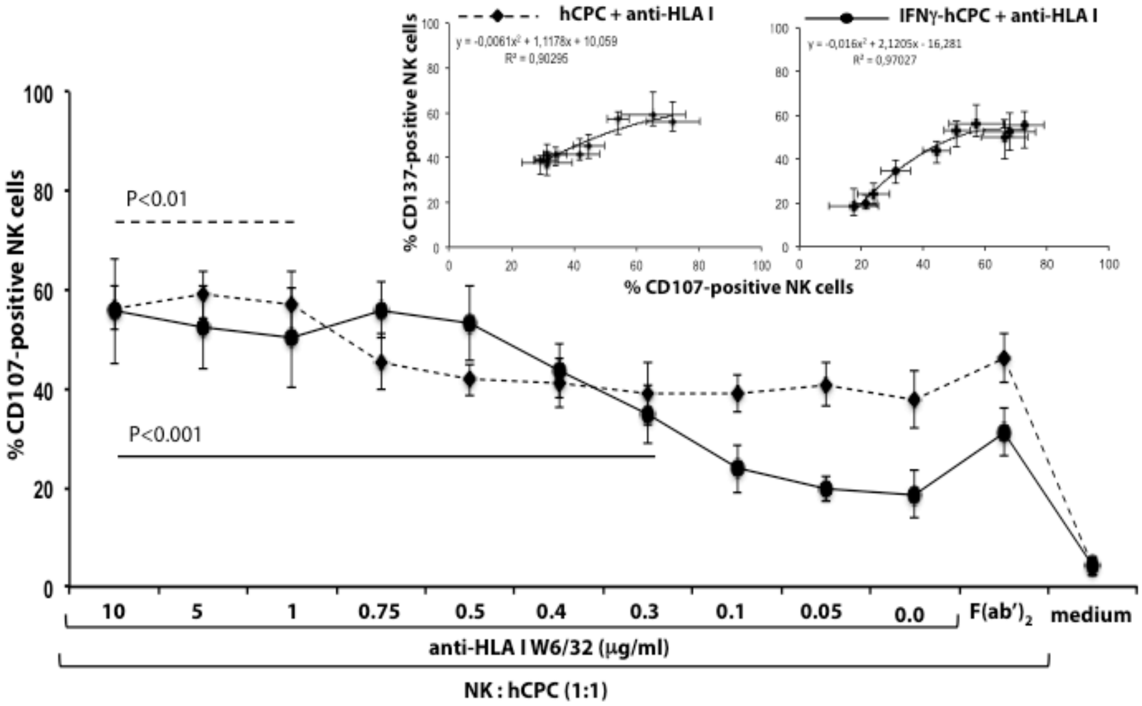

Supplementary Figure 3

Anti-HLA antibody-induced CD107a expression is binding strength-dependent. IL-15-activated NK cells were cultured alone (medium) or with hCPC or IFNγ-hCPC (n=6) in the presence of declining concentrations of anti-HLA I W6/32 or 10mg/ml of W6/32-F(ab')$_2$. % CD107a-positive NK cells determined by flow cytometry. % CD107a-positive NK cells observed for each antibody concentration in hCPC or IFNγ-hCPC plotted as function of respective MFIs (imbedded curves). Results are presented as mean values ± SD from three different experiments done with each hCPC. Statistical analyses were performed using One-Way Analysis of Variance (ANOVA)-Kruskal–Wallis test-dunn's multiple comparison (GraphPadPrism Software). *P*<0.01 and *P*<0.001 compared to NK+hCPC alone.

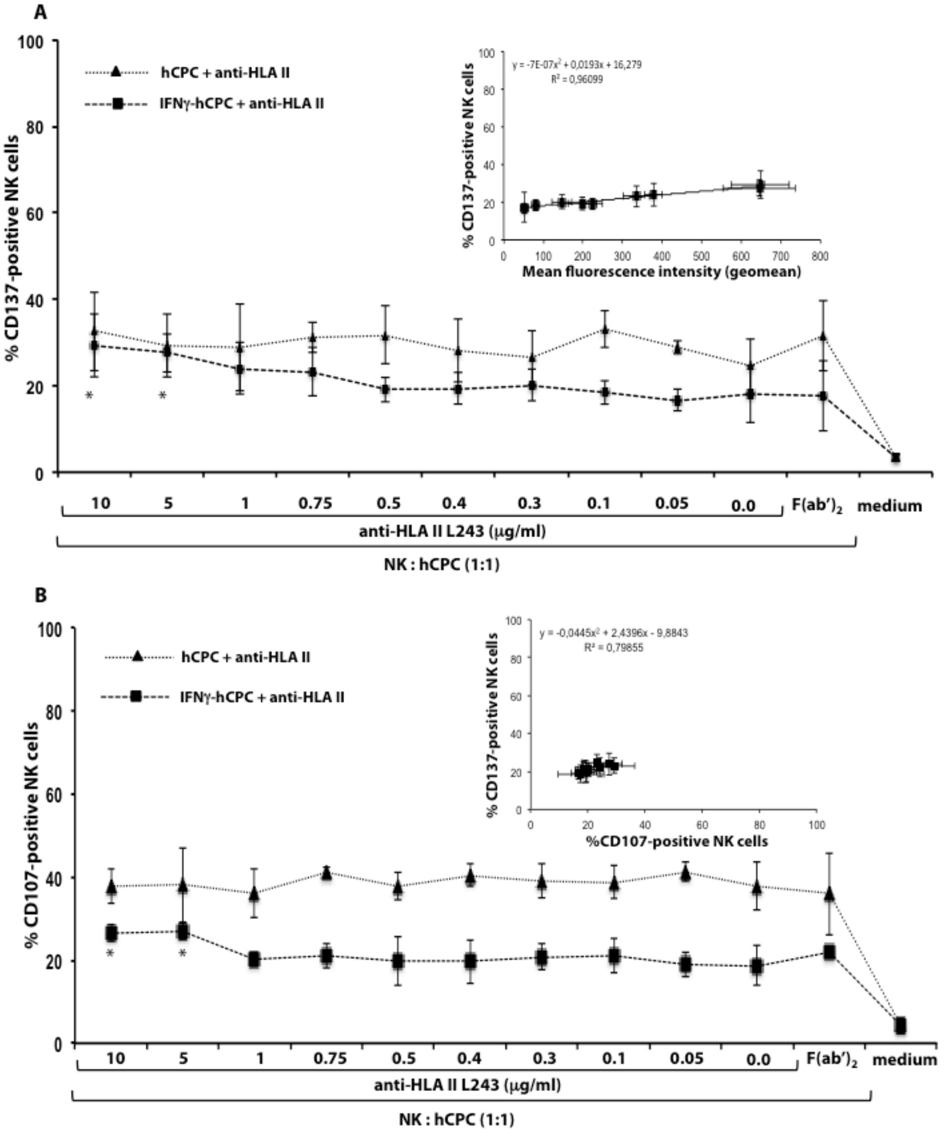

Supplementary Figure 4

Anti-HLA II modestly increases the expression of CD137 and CD107a. IL-15-activated NK cells were cultured alone (medium) or with hCPC or IFNg-hCPC (n=6) in the presence of declining concentrations of anti-HLA II L243 or 10mg/ml of L243-F(ab')$_2$. **(A)** % CD137-positive NK cells determined by flow cytometry. % CD137-positive NK cells observed for each antibody concentration on IFNg-hCPC plotted as function of respective MFIs (**A** imbedded curves). **(B)** % CD107a-positive NK cells determined by flow cytometry. % CD107a-positive NK cells observed for each antibody concentration on IFNg-hCPC plotted as function of respective MFIs (imbedded curves). Results are presentenced as mean values ± SD from three different experiments done with each hCPC. Statistical analyses were performed using One-Way Analysis of Variance (ANOVA)-Kruskal–Wallis test-dunn's multiple comparison (GraphPadPrism Software). **P*<0.05 compared to complement NK+hCPC.

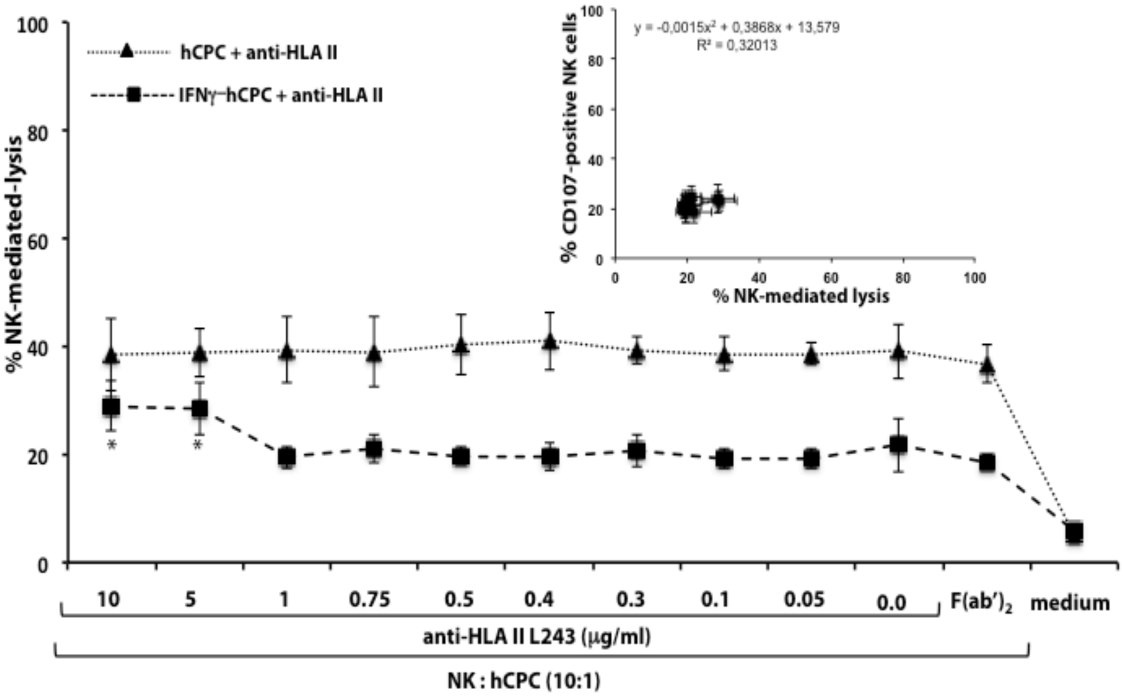

Supplementary Figure 5

Anti-HLA-II induces modest ADCC. IL-15-activated NK cells were cultured alone (medium) or with hCPC or IFNγ-hCPC (n=6) in the presence of declining concentrations of anti-HLA-II L243 or 10µg/ml of L243-F(ab')$_2$. % NK cell-mediated lysis evaluated as percentage of 7AAD-positive hCPC or IFNγ-hCPC. % CD107a-positive NK cells plotted as function of % NK-mediated lysis (imbedded curve). Results are presentenced as mean values±SD from three different experiments done with each hCPC. *P<0.05 compared to complement NK+hCPC. Statistical analyses were performed using One-Way Analysis of Variance (ANOVA)-Kruskal–Wallis test-dunn's multiple comparison (GraphPadPrism Software).

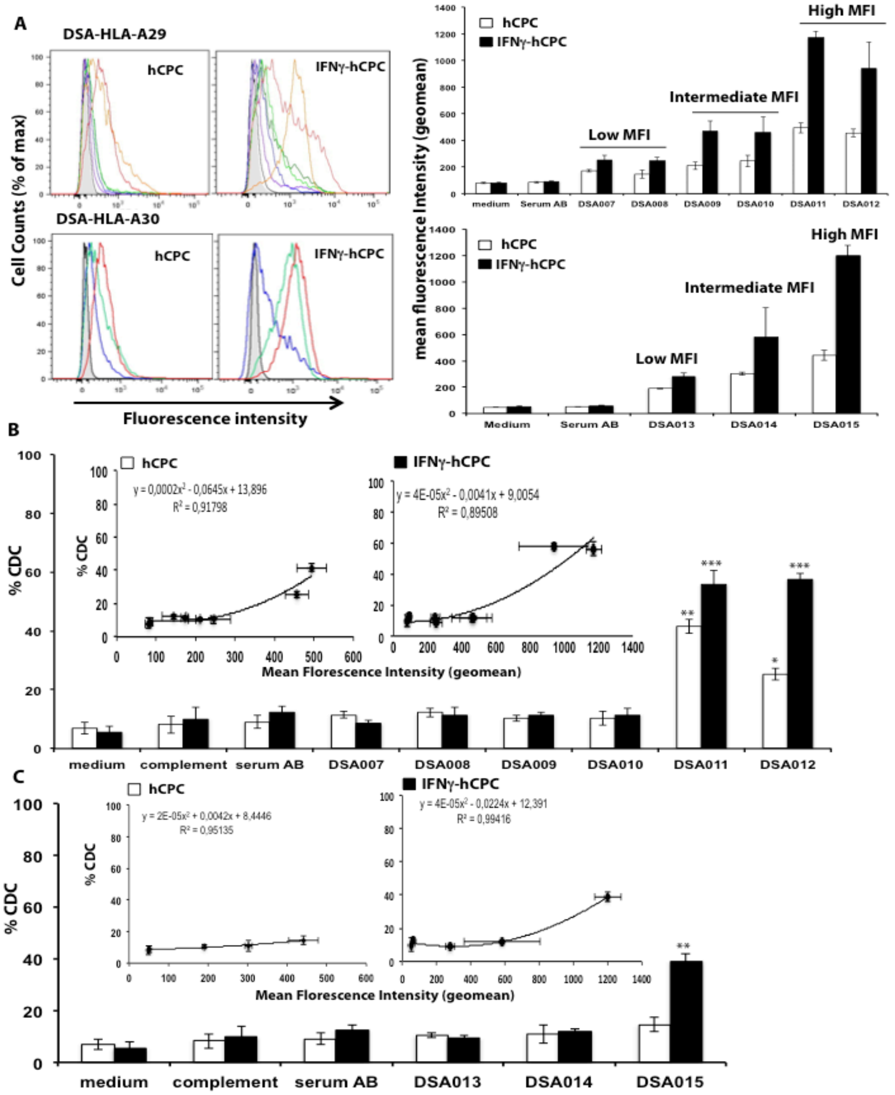

Supplementary Figure 6

DSA-HLA I-A29 and –A30 induce CDC against hCPC. DSA-HLA-I-A29 or -A30 sera (n=6 and 3, respectively) were incubated with HLA-A29 or A30-positive hCPC or IFNγ-hCPC, and their reactivity was determined as MFI by flow cytometry. **(A)** Representative histograms of A29- and A30-positive hCPC; respectively. A29: DSA007 (blue), DSA008 (purple), DSA009 (light green), DSA010 (dark green), DSA011 (red), DSA012 (orange). A30: DSA013 (blue), DSA014 (dark green), DSA015 (red). All are plotted against control serum AB (black) and medium alone (filled gray). Mean MFI (geomean) values±SD from four different experiments of each hCPC compared to serum AB and medium controls are shown in the right panel. **(B)** HLA I-A29-positive **(C)** A30-positive hCPC or IFNγ-hCPC were cultured alone, with control serum AB, or with the DSA-HLA I-A29 or –A30 sera, respectively, in the presence or absence of complement, then the capacity of DSA-HLA-I sera to induce CDC was evaluated by flow cytometry as percentage of 7AAD-positive hCPC. Results are presented as mean values±SD from three different experiments. % CDC induced by DSA-HLA-I-A29 (B imbedded curves) and –A30 (C imbedded curves) sera in hCPC or IFNg-hCPC were plotted as function of respective MFIs. Statistical analyses were performed using Mann–Whitney test for non-paired groups. **P*<0.05, ***P*<0.01, ****P*<0.001 compared to hCPC in the presence of complement alone.

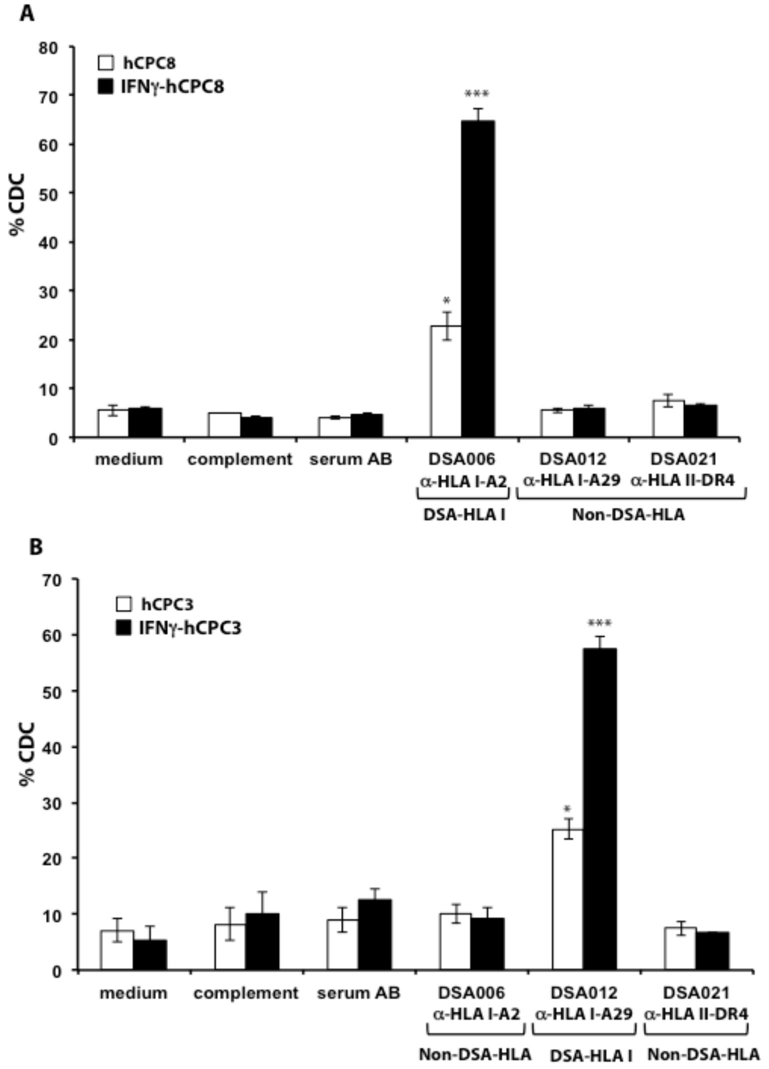

Supplementary Figure 7

Non-DSA-HLA are not cytotoxic to hCPC. A) HLA-A2-positive hCPC8 or IFNγ-hCPC8 (HLA-A2/24, HLA-B15/35, HLA-C01/04, HLA-DR14/08), were cultured alone (medium), with control serum AB, with DSA-HLA-I-A2 (DSA006) containing hCPC8-specific anti(a)-HLA-I-A2 antibodies, or with two sera DSA-HLA I-A29 (DSA012), or DSA-HLA-II-DR4 (DSA021) containing hCPC8-nonspecfic anti(a)-HLA-I-A29 and anti(a)-HLA-II-DR4 antibodies as non-DSA-HLA, in the presence or absence of complement. The capacity of the sera to induce CDC was evaluated by flow cytometry as percentage of 7AAD-positive hCPC. **(B)** HLA-A29-positive hCPC3 or IFNγ-hCPC3 (HLA-A29/29, HLA-B50/57, HLA-C03/16, HLA-DR07/13), were cultured alone (medium), with control serum AB, with DSA-HLA-I-A2 (DSA006) containing hCPC3-nonspecific anti(α)-HLA I-A2 as non-DSA-HLA sera, with DSA-HLA-I-A29 (DSA012) containing hCPC3-specific anti(a)-HLA-I-A29, or with DSA-HLA-II-DR4 (DSA021) containing hCPC3-nonspecific anti(a)-HLA-II-DR4 sera also as non-DSA-HLA, in the presence or absence of complement. The capacity of the sera to induce CDC was evaluated by flow cytometry as percentage of 7AAD-positive hCPC. Results are presented as mean values ± SD from three different experiments. Statistical analyses were performed using Mann–Whitney test for non-paired groups. **P*<0.05, ****P*<0.001 compared to hCPC in the presence of complement alone.

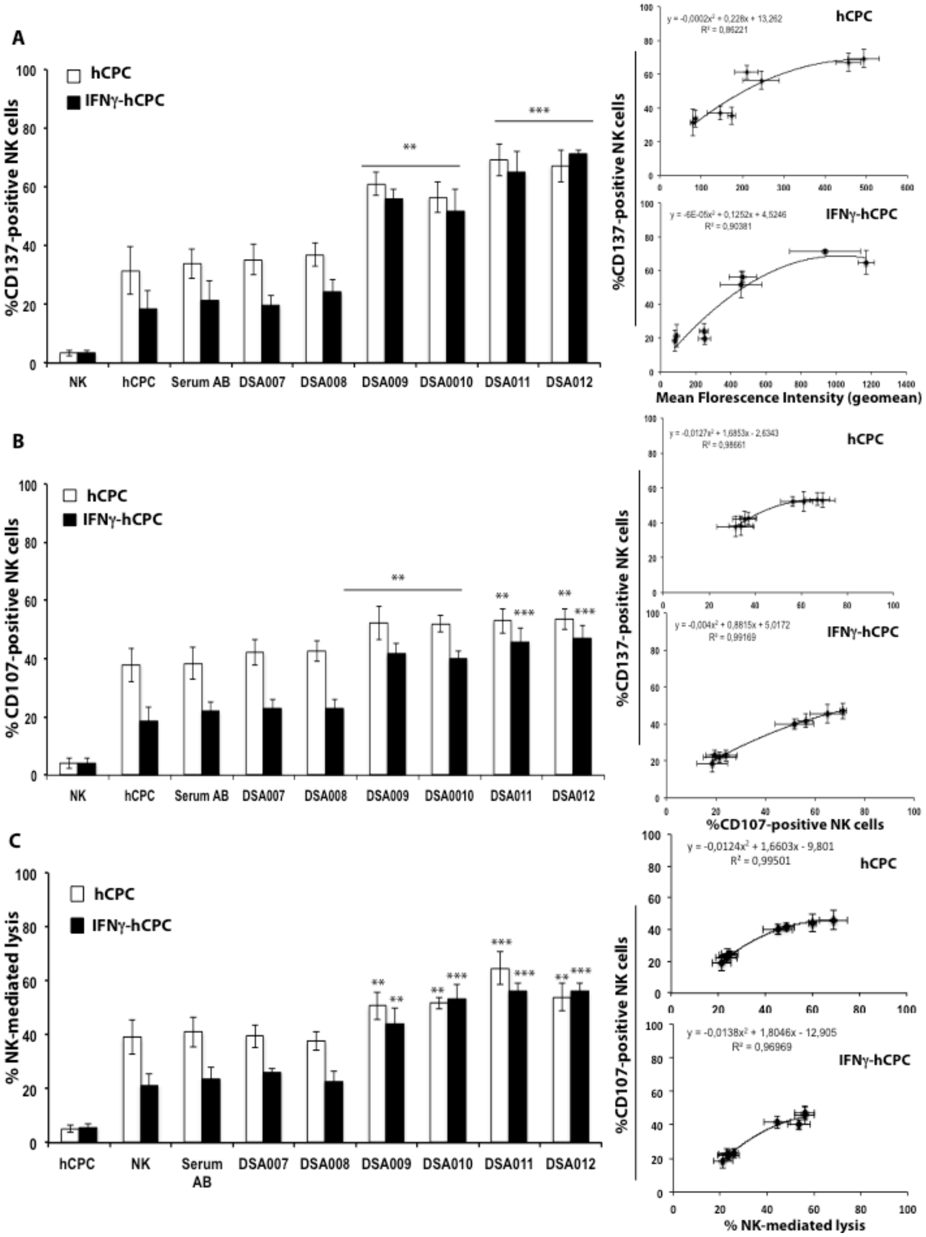

Supplementary Figure 8

DSA-HLA-I-A29 of high and intermediate binding strength induce ADCC. IL-15-activated NK cells were cultured alone or with HLA-A29 positive hCPC or IFNγ-hCPC in the presence of control serum AB or DSA-HLA I-A2 sera (DSA007-012). **(A)** % CD137-positive NK cells, **(B)** % CD107-positive NK cells, and **(C)** % NK-mediated lysis evaluated as % 7AAD-positive hCPC determined by flow cytometry. Results represent mean values±SD from four different experiments of each hCPC. The percentages of CD137-positive NK cells with hCPC or IFNγ-hCPC were plotted as function of respective MFIs of DSA-HLA I sera (upper right panel) or as function of % CD107-positive NK cells (middle right panel), the percentages of CD107-positive NK cells were plotted as function of % NK-mediated lysis (low right panel) for both hCPC and IFNγ-hCPC. Statistical analyses were performed using Mann–Whitney test for non-paired groups. ***P*<0.01, ****P*<0.001 compared to NK+hCPC.

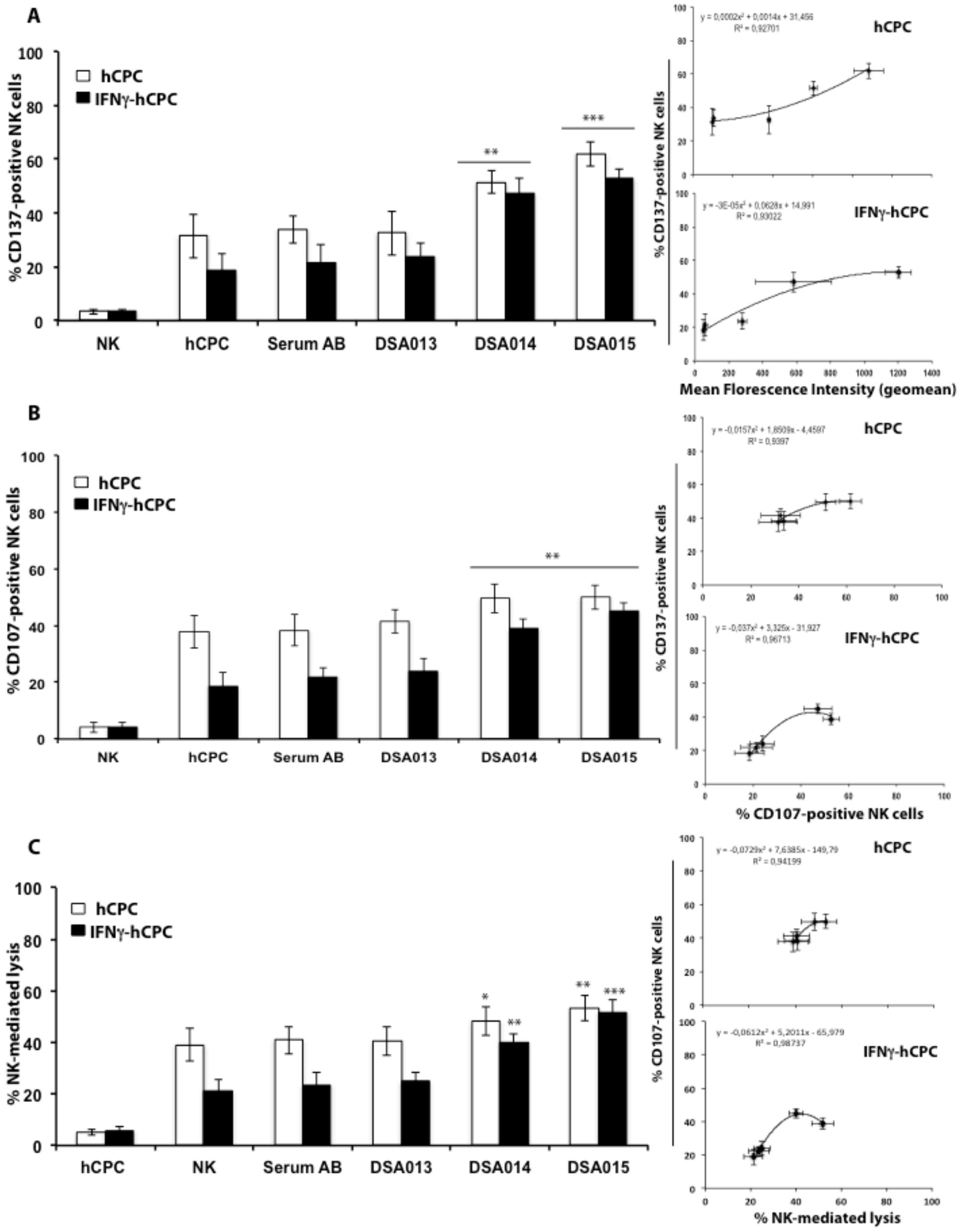

Supplementary Figure 9

DSA-HLA I-A30 of high and intermediate binding strength induce ADCC. IL-15-activated NK cells were cultured alone or with HLA-A30 positive hCPC or IFNγ-hCPC in the presence of control serum AB or DSA-HLA I-A2 sera (DSA013-015). **(A)** % CD137-positive NK cells, **(B)** % CD107-positive NK cells, and **(C)** % NK-mediated lysis evaluated as % 7AAD-positive hCPC determined by flow cytometry. Results represent mean values±SD from four different experiments of each hCPC. The percentages of CD137-positive NK cells with hCPC or IFNγ-hCPC were plotted as function of respective MFIs of DSA-HLA I sera (upper right panel) or as function of % CD107-positive NK cells (middle right panel), the percentages of CD107-positive NK cells were plotted as function of % NK-mediated lysis (low right panel) for both hCPC and IFNγ-hCPC. Statistical analyses were performed using Mann–Whitney test for non-paired groups. *P<0.05, **P<0.01, ***P<0.001 compared to NK+hCPC.

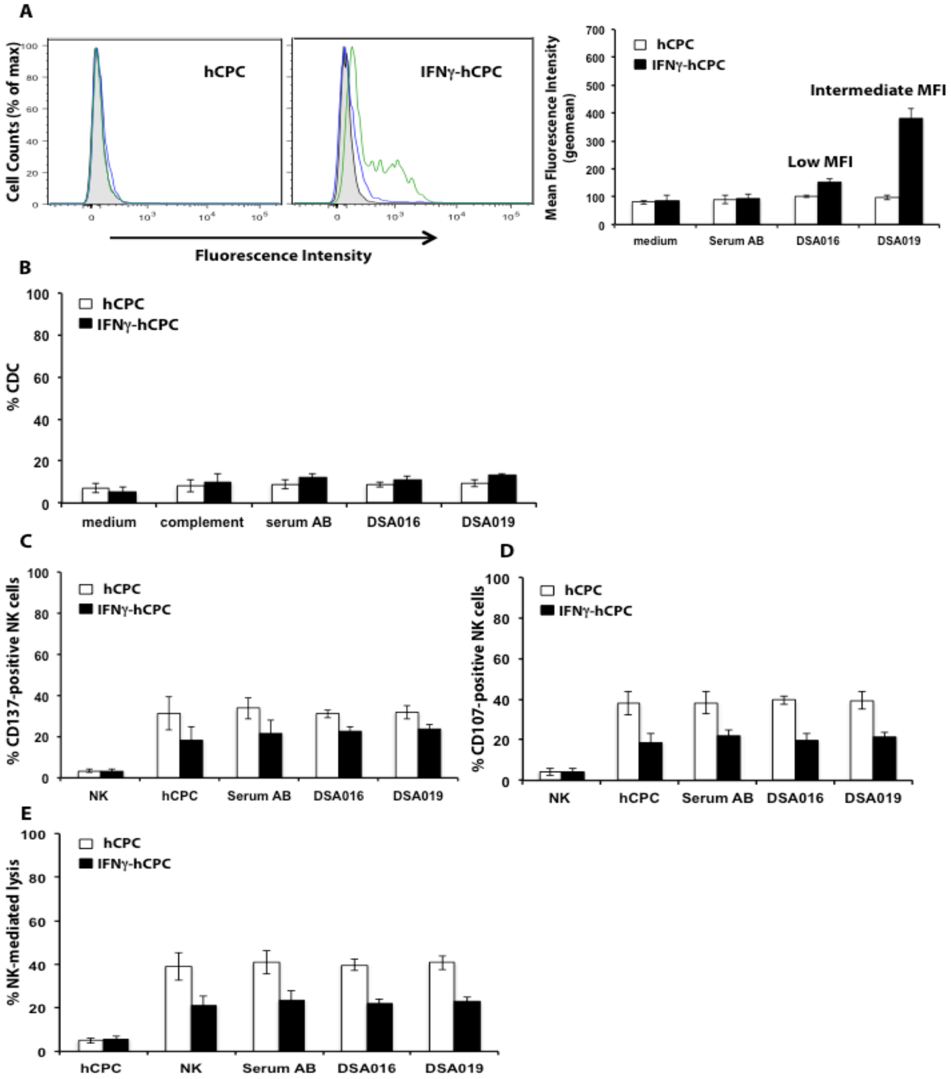

Supplementary Figure 10

DSA-HLA-II-DR1 sera do not induce antibody-mediated cytotoxicity. DSA-HLA-II-DR1 sera (n=2) were incubated with HLA-DR1-positive hCPC or IFNγ-hCPC (n=2) then their reactivity was determined as MFI by flow cytometry. **(A)** Left panel showing representative histograms of DSA016 (blue) and DSA019 (green) interactions against control serum AB (black) and medium alone (filled gray). Mean MFI (geomean) values±SD from three different experiments of each hCPC compared to serum AB and medium controls (right panel). **(B)** HLA-DR1-positive hCPC or IFNγ-hCPC (n=2) were cultured alone, with control serum AB, or with DSA-HLA II-DR1 sera (DSA016, 019), in the presence or absence of complement, then their capacity to induce CDC was evaluated by flow cytometry as percentage of 7AAD-positive hCPC. Results are presented as mean values±SD from four different experiments of each hCPC. (**C-E)** IL-15-activated NK cells were cultured alone or with HLA-DR1-positive hCPC or IFNγ-hCPC (n=2) in the presence of control serum AB or DSA-HLA II-DR1 sera. **(C)** % CD137-positive NK cells, **(D)** % CD107-positive NK cells and **(E)** % NK-mediated lysis evaluated as % 7AAD-positive hCPC determined by flow cytometry. Results represent mean values±SD from three different experiments of each hCPC. Statistical analyses were performed using Mann–Whitney test for non-paired groups.

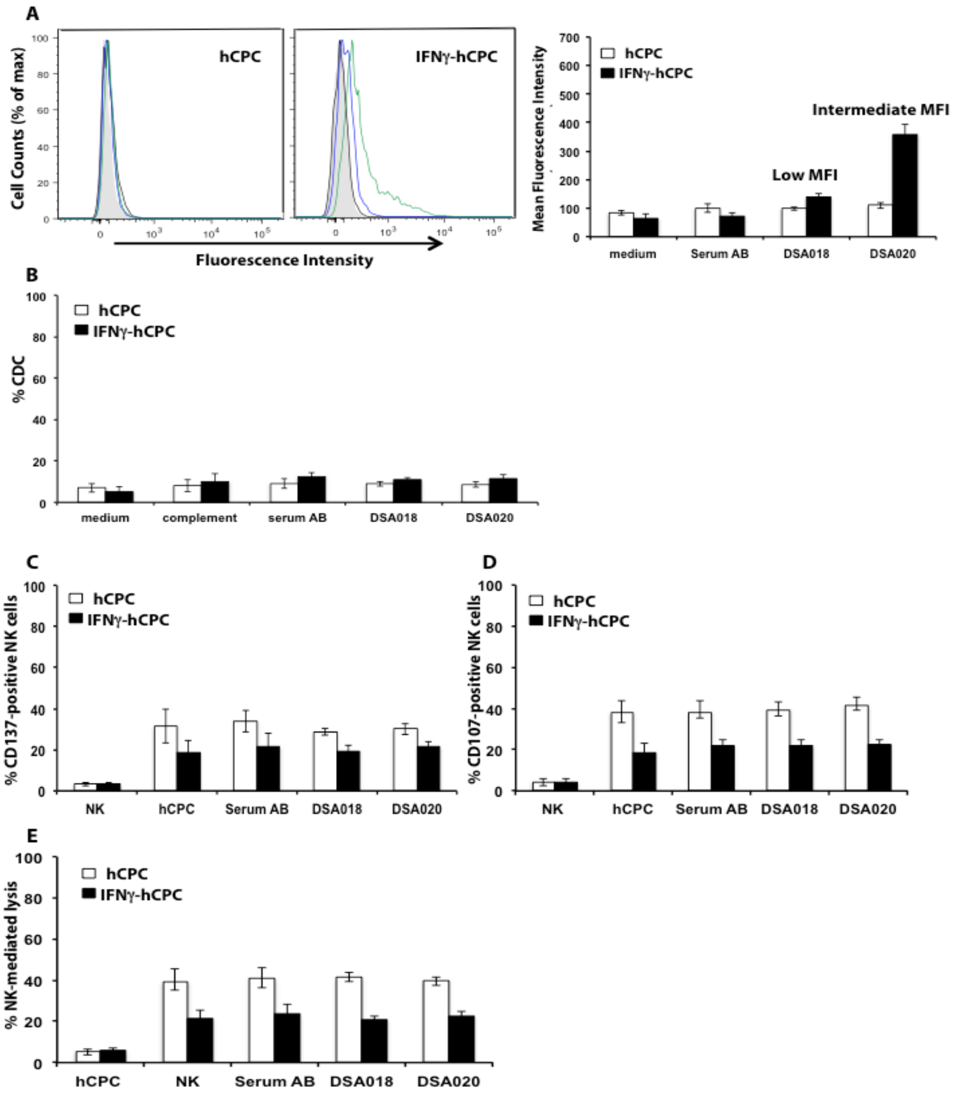

Supplementary Figure 11

DSA-HLA-II-DR13 sera do not induce antibody-mediated cytotoxicity. DSA-HLA-II-DR13 sera (n=2) were incubated with HLA-DR13-positive hCPC or IFNγ-hCPC (n=2) then their reactivity was determined as MFI by flow cytometry. **(A)** Left panel showing representative histograms of DSA018 (blue) and DSA020 (green) interactions against control serum AB (black) and medium alone (filled gray). Mean MFI (geomean) values±SD from three different experiments of each hCPC compared to serum AB and medium controls (right panel). **(B)** HLA-DR13-positive hCPC or IFNγ-hCPC (n=2) were cultured alone, with control serum AB, or with DSA-HLA II-DR13 sera (DSA018, 020), in the presence or absence of complement, then their capacity to induce CDC was evaluated by flow cytometry as percentage of 7AAD-positive hCPC. Results are presented as mean values±SD from four different experiments of each hCPC. (**C-E)** IL-15-activated NK cells were cultured alone or with HLA-DR1-positive hCPC or IFNγ-hCPC (n=2) in the presence of control serum AB or DSA-HLA II-DR1 sera. **(C)** % CD137-positive NK cells, **(D)** % CD107-positive NK cells and **(E)** % NK-mediated lysis evaluated as % 7AAD-positive hCPC determined by flow cytometry. Results represent mean values ± SD from three different experiments of each hCPC. Statistical analyses were performed using Mann–Whitney test for non-paired groups.

Supplementary Table 1: hCPC HLA class I and HLA class II genotyping
hCPC were genotyped for HLA I and HLA II to monitor their interactions with DSA-HLA

	HLA-I						HLA-II	
	HLA-A		HLA-B		HLA-C		DRB1	
hCPC 3	29	29	50	57	03	16	0701	1302
hCPC 6	02	02	44	44	05	16	13	13
hCPC 7	02	32	35	44	04	16	04	04
hCPC 8	02	24	15	35	01	04	14	08
hCPC 9	02	03	15	39	12	03	01	04
hCPC 10	24	30	14	18	02	05	01	03

Supplementary Table 2: Binding strength of DSA-HLA-I and DSA-HLA-II to hCPC and IFNγ-hCPC

Serum number	Specificity	Luminex-based MFI	Cytometry-based MFI (hCPC)	Cytometry-based MFI (IFNg-hCPC)
DSA001	HLA-I A2	3040	111	299
DSA002	HLA-I A2	2000	110	342
DSA003	HLA-I A2	7000	160	573
DSA004	HLA-I A2	7500	179	630
DSA005	HLA-I A2	20951	536	1650
DSA006	HLA-I A2	17654	515	1251
DSA007	HLA-I A29	2500	180	275
DSA008	HLA-I A29	1936	167	264
DSA 009	HLA-I A29	6156	230	468
DSA 010	HLA-I A29	5668	276	458
DSA 011	HLA-I A29	19710	521	1172
DSA 012	HLA-I A29	16210	478	973
DSA 013	HLA-I A30	2800	190	279
DSA 014	HLA-I A30	6257	302	583
DSA 015	HLA-I A30	13050	441	1202
DSA 016	HLA-II DR1	1025	101	153
DSA 017	HLA-II DR4	1001	75	149
DSA 018	HLA-II DR13	1001	99	140
DSA 019	HLA-II DR1	6709	96	380
DSA 020	HLA-II DR13	5099	110	359
DSA 021	HLA-II DR4	16471	90	513

Supplementary Table 3: List of Antibodies

Antibody	Clone	Fluorochrome	Distributor
HLA-I	W6/32	-	BIOTEM
HLA-I F(ab')^2	W6/32	-	BIOTEM
HLA-II (HLA-DR)	L243	-	BIOTEM
HLA-II F(ab')^2	L243	-	BIOTEM
HLA-II (HLA-DQ)	33.1	-	Affinity purified
HLA-II (HLA-DP)	B27/2	-	Affinity purified
CD107	H4A3	PE	BD Pharmingen
CD 137	4B4-1	Pecy5	BD Pharmingen
CD3	SK7	APC-H7	BD Biosciences
CD56	B159	Pecy7	BD Biosciences
Goat anti-mouse IgG	polyclonal	PE	Sigma
Rabbit anti-human IgG Fc	polyclonal	FITC	Pierce Antibody

Supplementary Methods

Human Cardiac stem/progenitor cells (hCPC) culture: Human cardiac biopsies were obtained from patients (n=6) undergoing an open-chest surgery, usually for valve replacement, after signed informed consent. The ethical committees of "Hospital 12 de Octubre", "Fundación Jiménez Díaz", (Madrid) and "Complejo Hospitalario de Navarra" (Pamplona) - Spain have approved the project. Cardiac stem/progenitor cells (hCPC) were obtained from the right atria appendage after immune-depletion of CD45-positive cells and immune-selection of CD117 (c-kit). The cells were expanded, characterized for genetic stability, and then cryopreserved. Upon thawing, cells are grown and maintained in a combination of DMEM/F12 and Neurobasal (1:1) medium supplemented with 10% fetal bovine serum embryonic stem cell qualified (FBS-ESCq), L-Glutamine (2 mM), Penicillin-Streptomycin (100U/mL and 100µg/mL), bFGF (10ng/mL), Insulin-Transferrin-Selenium (ITS), B27 (1X), N2 (1X), β-mercaptoethanol (50µM) (Invitrogen, Saint-Aubin, France), IGF-II (30ng/mL) and EGF (20ng/mL) (Peprotech, Neuilly-sur-Seine, France) at 3% O2 atmosphere mimicking pathophysiological conditions. hCPC have been fully characterized in our previous report [1] as stem cells with mixed phenotype expressing pluripotency factors oct4, sox2 and nanog as well as early cardiac lineage transcription factors GATA-4, MEF2C, Nkx2.5. hCPC are able to form cardiospheres in non-adherent culture conditions and differentiate into the three principal cardiac lineages *in vitro*. Under inflammatory conditions, mimicked by IFNγ stimulation (500IU/ml), hCPC over-express Human Leukocyte Antigens (HLA) class I and class II but conserve their morphology and pluripotency[1] and (Supplementary Fig. 1). Injection of hCPC in Nude rats after infarct significantly improves cardiac function [1]. hCPC from all the donors (n=6) were genotyped for HLA using routine standard techniques at the Laboratory of Immunology and Histocompatibility, Saint Louis Hospital, Paris, France (Table 1). All assays were performed with passages 3-7 hCPC at 80–90% confluence.

Monoclonal anti-HLA antibodies and allosera: Mouse anti-human HLA-I-A, -B, -C (W6/32), anti-human HLA-DR (L243) monoclonal antibodies (mAb), anti-HLA-I $F(ab')_2$ and anti-HLA-DR $F(ab')_2$ were obtained from Biotem France. Cryopreserved allosera from heart transplantation patients (pre-transplantation) containing panel reactive antibodies (PRA) – allosera containing HLA antibodies, were provided by the Laboratory of Immunology and Histocompatibility, Saint Louis Hospital, Paris, France, and used in accordance with local institutional regulations with the approval of the local ethic committee. Allosera were screened to identify the specificity of their HLA antibodies by single-antigen flow beads Luminex assay as described[2]. Briefly, this assay uses sets of 97 beads (class I) and 86 beads (class II). Each bead is coated with a single HLA glycoprotein, which allows for the precise identification of Ab specificity. The presence of Abs is detected using a goat anti-human IgG coupled with phycoerythrin. The fluorescence of each bead is detected by a reader (LABscan; Luminex, Austin, TX) and recorded as the MFI. Beads showing normalized MFI greater than 500, are considered positive. Sera containing DSA-HLA-I against HLA-A and DSA-HLA-II against HLA-DR were considered positive when their binding strength by Luminex is greater than 500 (Table 2). Human AB serum was obtained from the Institut de biotechnologies - Jacques Boy (Reims, France) and used as negative control.

Natural Killer (NK) cells purification: Peripheral blood mononuclear cells (PBMC) were prepared from blood samples of different healthy donors (n=10) as well as from patients with MI (n=3) by centrifugation on a Ficoll-Hypaque density gradient. The PBMC from all the donors were genotyped for HLA at the Laboratory of Immunology and

Histocompatibility, Saint Louis Hospital, Paris, France. Donors signed an informed consent approved by human ethics committee “Comité consultatif pour la protection des personnes dans les recherches biomédicales” - Saint Louis Hospital, Paris, France), and the study has been approved by the institution. NK cells were isolated from PBMC using negative selection immunomagnetic cell sorting (Miltenyi Biotech, Bergish Gladbach, Germany) according to manufacturer's instructions. Briefly, a cocktail of antibodies specific for CD3, CD4, CD8, CD19, CD20, and CD14 were used to retain T and B lymphocytes as well as monocytes leaving NK cells untouched. All experiments implicating NK cells were conducted with freshly isolated cells activated overnight with recombinant human IL15 (50ng/mL) (Immunotools, Friesoythe, Germany) in RPMI medium supplemented with 10% FBS, to ensure expression of all NK cell activating receptors and their optimum function[3] and were performed in allogeneic settings.

Immune phenotyping: To determine the level of HLA-I and HLA-II expression on hCPC, cells were incubated with 10μg/ml of anti-HLA I W6/32 or anti-HLA II (anti-HLA-DR L243, anti-HLA-DQ 33.1, or anti-HLA-DP B7/21) mAbs for 30 min in 100μl PBS at 4°C. Cells were then washed and incubated for another 30 min with PE-conjugated goat anti-mouse IgG secondary antibody then washed. Cells were acquired using Canto II flow cytometer (BD Biosciences, le Pont-de-Claix, France) and analyzed using either the BD FACS Diva or FlowJo software (Celeza, Olten, Switzerland). The expression levels of HLA-I or -II antigens are presented as geometric mean of fluorescence intensity (MFI).

Flow Cytometry-based binding assay: A cytometry-based assay tailored for hCPC was developed to determine the “binding strength” of different concentrations of anti-HLA-I, anti-HLA-II or DSA-HLA. Cells were incubated for 1h at 4°C in 100μl PBS containing different concentrations of mAbs or with 10μL pure alloserum containing anti-HLA antibodies (DSA-HLA). Cells were then washed and re-incubated for another 1h at 4°C in 100μL PBS containing 1μL of PE-conjugated goat anti-mouse IgG (for mAbs) or with FITC-conjugated rabbit anti-human IgG (for allosera) secondary antibodies. Cells were acquired using the Canto II flow cytometer (BD Biosciences, le Pont-de-Claix, France) and analyzed using either the BD FACS Diva or FlowJo software (Celeza, Olten, Switzerland). The binding strength is presented as geometric mean of fluorescence intensity (MFI).

Complement-Dependent-Cytotoxicity (CDC) – hCPC-tailored cross-match: hCPC or IFNγ-treated hCPC were incubated with anti-HLA-I or anti-HLA-II mAbs at different concentrations or with allosera containing HLA antibodies for 1h at room temperature (RT). Cells were then washed and re-incubated with 50μL of pure complement (Bio-Rad AbD Serotec Ltd., Oxford – UK) for 30 min at RT. After washing the percentage of lysed hCPC was assessed by 7AAD (BD Bioscience) staining and Canto II flow cytometer and analyzed by BD FACS Diva software.

Antibody-dependent cell-mediated cytotoxicity (ADCC):
1) CD137 expression and degranulation of NK cells: Freshly-isolated NK cells were activated overnight with IL-15 (50ng/ml) then co-cultured for another overnight with hCPC or IFNγ-treated hCPC at ratio of 1:1 in the presence or the absence of different concentrations of anti-HLA mAbs or allosera containing HLA antibodies at 37°C in 3% O2 atmosphere. IFNγ-hCPC were washed before co-culture with NK cells to avoid any stimulation of the immune cells by residual IFN-γ. NK degranulation was determined by adding the anti-CD107a mAb at the beginning of the co-cultures whereas anti-CD137

was added at the end for another 1h to determine the engagement of CD16 on NK cells. Cells were then washed and acquired on Canto II flow cytometer and NK cells were gated as $CD3^-CD56^+$ cells. The percentage of CD107a-positive and CD137-positive cells was then determined and analyzed by BD FACS Diva software.

2) NK cell-mediated lysis: hCPC or IFNγ-hCPC were labeled with Carboxyfluorescein succinimidyl ester (CFSE) (2.5μM) for 10min at 37°C, then washed 3 times with RPMI 10% FBS. CFSE-labeled hCPC were then co-cultured with IL-15-activated NK cells at a NK : hCPC ratio of 10:1 in the presence or absence of various concentrations of mAbs or allosera, for 4h at 37°C in 3% O2 atmosphere. After washing, cells were acquired on Canto II flow cytometer and the percentage of CFSE-labeled 7-AAD-positive hCPC or IFNg-hCPC was determined and analyzed by BD FACS Diva software.

Statistical analysis: Statistical analyses were performed using Mann–Whitney test for non-paired groups, paired Student's t-test for paired groups and One-Way Analysis of Variance (ANOVA)-Kruskal–Wallis test-dunn's multiple comparison for multiple comparison (GraphPadPrism Software). Data are expressed as mean value±SD, P-values < 0.05 were considered significant. **P*<0.05, ***P*<0.01, ****P*<0.001.

References

1. Lauden L, Boukouaci W, Borlado LR, Lopez IP, Sepulveda P, Tamouza R, Charron D, Al-Daccak R. Allogenicity of human cardiac stem/progenitor cells orchestrated by programmed death ligand 1. *Circ Res*. 2013;112:451-464
2. Brugiere O, Suberbielle C, Thabut G, Lhuillier E, Dauriat G, Metivier AC, Gautreau C, Charron D, Mal H, Parquin F, Stern M. Lung transplantation in patients with pretransplantation donor-specific antibodies detected by luminex assay. *Transplantation*. 2013;95:761-765
3. Carson WE, Giri JG, Lindemann MJ, Linett ML, Ahdieh M, Paxton R, Anderson D, Eisenmann J, Grabstein K, Caligiuri MA. Interleukin (il) 15 is a novel cytokine that activates human natural killer cells via components of the il-2 receptor. *J Exp Med*. 1994;180:1395-1403
